# Experimental induction of state rumination: A study evaluating the efficacy of goal-cueing task in different experimental settings

**DOI:** 10.1371/journal.pone.0288450

**Published:** 2023-11-22

**Authors:** Alena Michel-Kröhler, Michèle Wessa, Stefan Berti

**Affiliations:** 1 Department of Clinical Psychology and Neuropsychology, Institute for Psychology, Johannes Gutenberg-University Mainz, Mainz, Germany; 2 Leibniz Institute for Resilience Research (LIR), Mainz, Germany; University of Hertfordshire, UNITED KINGDOM

## Abstract

Based on previous studies, the present four experiments (total *N* = 468) aimed at investigating the effectivity of rumination induction in different experimental settings. We were particularly interested in rumination in the context of individual goal achievement and tested whether an instruction that referred to unresolved goals had a direct observable effect on state rumination. For this purpose, participants were asked to identify, evaluate, and focus on a personally relevant goal that was previously unresolved and still bothered them. In Experiment 1a to 1c, we compared three different modifications of the unresolved condition with shortened instructions with the elaborated unresolved condition and an additional control condition that did not refer to goals. In general, the results were mixed, but basically confirmed the effectiveness of the method used. Finally, in Experiment 2, we compared the two most promising versions of the unresolved condition and, by adding a goal-related control condition, we examined which control condition was best suited to maximize effects related to state rumination in future research. Results of various mixed ANOVAs demonstrated that a shortened version (in terms of shortened audio instructions) of the unresolved condition could be used as well as the original unresolved condition to induce reliable state rumination. The significance of the effects obtained with this method for real-life applications as well as approaches for future research are discussed.

## Introduction

Rumination, defined as repetitive, intrusive thoughts, can affect different cognitive processes, and has the potential to impair performance, psychological wellbeing, or mental health [1,2]. Despite increasing research in this area, specific mechanisms underlying these negative effects are not completely understood. One way to gain detailed insight into the effects of rumination as well as the mechanisms that cause these effects, is to induce ruminative thoughts within an experimental design so that a direct measurement of the potential effects of rumination on selected outcome variables is possible. However, to the best of our knowledge evaluation of rumination induction tasks outside of a clinical context is lacking. Here, we report results of four experiments in which we aimed at evaluating the effectivity of a procedure to induce rumination (namely, the goal-cueing task [3]) in different experimental settings, with the goal of enabling its application in and outside the laboratory. In contrast to other approaches, we focus on a non-clinical setting because the overall aim of the study is to establish an effective procedure for rumination induction within a broad range of rumination research. In the following, we will first describe the theoretical background of the goal-cueing task to motivate the application of this specific procedure. Second, we will give an overview of the four experiments performed within this study.

### Relevance of goals in our daily lives and the Goal-Progress Theory

Goals structure our daily actions and serve as the main motivation for pursuing our careers, our studies, our leisure activities such as sports, and various other activities in our private lives. In doing so, we try to pursue various goals from different areas at the same time ("providing a good presentation", "keeping physically fit", "being a good friend", etc.) [4]. However, what happens when we fail to achieve a goal that we really want to achieve, or when we continue to hold on to a goal even though we seem not capable of achieving it [5]? A discrepancy occurs that implies dissatisfaction with the current state and a desire to achieve a goal or specific outcome [6]. As a result, negative thoughts and feelings can arise that revolve around the failed goal achievement, and if persistent and recurring, can lead to rumination [5].

The Goal Progress Theory (GPT) by Martin and Tesser [4] provides a detailed account of goal discrepancies as a central mechanism of rumination. GPT states that people’s thoughts and actions are guided by individual, conscious or unconscious goals, which thus play an important role in the self-regulation of behavior [7]. In addition, a lack of progress (more or less) toward an individual goal is likely to trigger rumination, which persists until the discrepancy is resolved either by restoring goal progress or by moving away from the goal [4]. It is worth noting that rumination does not necessarily have to be dysfunctional. It can also be adaptive and functional in certain circumstances to highlight unresolved issues and then address them as progress is made in reducing the discrepancy [1,8]. However, rumination often does not help to reduce goal discrepancy or to disengage from the goal even when there is no prospect of success. This is especially the case when individuals are unable to reprioritize or identify alternative ways to achieve the goal [4]. Thus, rumination can be seen mainly as an unsuccessful problem-solving attempt that is unhelpful and causes a persistent goal discrepancy [1,4]. Nevertheless, individuals continue to ruminate in some situations, believing that this gives them an advantage or brings them closer to their goal [9–11]. Therefore, first, it is important to understand the thoughts associated with the individual goals and how they affect individuals’ well-being and daily performance. Second, it is advantageous if individuals adopt a proactive attitude after setbacks or failures and sustain capabilities that contribute to the improvement of the situation instead of remaining in the situation that has occurred [12–14].

To date, some studies have linked problematic goal attainment with rumination: For instance, results of an experience-sampling study [15] revealed that low goal success and high goal importance was associated with high levels of negative affect, and that this interaction was marginally significant for ruminative self-focus. In addition, Huffziger and colleagues [16,17] demonstrated that induced rumination in an everyday life application immediately deteriorated mood-related valence and calmness and was linked to stronger reductions in positive mood. Moreover, Krys [18] showed in a two-week diary study that rumination exerted a negative indirect effect on subjective problem solving via perceived stress and negative mood. In contrast, results of another study [8] demonstrated that goal-directed rumination has positive effects on academic performance if negative effects of psychological distress were simultaneously accounted for. However, goal-directed rumination per se was not related to academic performance [8]. Finally, one study from the context of sports [19] showed that athletes who fulfilled their goals at the end of a competitive season had lower rumination scores compared with athletes who did not. Overall, the results of the studies indicate that rumination can play a relevant role in different contexts during the individual goal achievement process. Moreover, these findings also suggest that goals, more precisely, the perceived goal discrepancy, can serve as a trigger for rumination especially if they are related to failure.

### Rumination and the goal-cueing task

Rumination and its consequences have attracted increasing theoretical and empirical interest in the past 30 years [20,21]. In this research process, rumination has been examined both as a habitual tendency toward repetitive and passive self-focus in response to depressed mood [1,22] and as a temporary cognitive response that is highly dependent on situational cues [4,20,23,24]. In this context, numerous scales have been developed to capture rumination as a trait (for an overview see Krys [25]), including the Rumination-Reflection Questionnaire (RRQ; [26]) and the Perseverative Thinking Questionnaire (PTQ; [27]). In contrast, there is no “gold standard” for measuring state rumination [23]. Numerous studies have used various state rumination measures with different psychometric properties and levels of validity, among which the Brief State Rumination Inventory (BSRI) can be considered the most established [23]. In addition, however, various experimental approaches have been developed with the aim of examining ruminative thoughts within an experimental study so that a direct measurement of the potential effects of rumination on selected outcome variables is possible [e.g., 28–31]. An effective possibility of inducing rumination is the response manipulation task, which was designed to influence the contents of participants’ thoughts by forcing them to focus their attention on emotion-, symptom- and self-focused thoughts (cf. [31], e.g.: “think about how active/passive you feel” or “think about what your feelings might mean”). The response manipulation task is well established, but limits rumination to a specific subset of thoughts related to mental health. A potential alternative to induce ruminative thoughts, which might be more common in everyday life, is the so-called goal-cueing task by Roberts and colleagues [3]. This task does not restrict or pre-define the area of ruminative thoughts by cueing specific feelings and personal attributes related to rumination. Instead, it focuses on concerns or problems during the individual goal achievement process. In this context, participants are free to choose the content so that a broader range of thoughts can be addressed, which occur more naturally in everyday situations. Ruminating can refer, for example, to a certain negative event and its influence on how the person has felt in recent weeks. It can also be thoughts about the fact that the comparison to other people in relation to a personal important aspect is not positive or one has the feeling to disappoint a person.

The goal-cueing task developed by Roberts and colleagues [3,32] consists of three steps, namely (1) identification of a problem in the individual goal achievement process, (2) evaluation of the identified problem, and (3) 10-minute goal focus period, which is predicted to elicit rumination. In addition, the task consists of two conditions, a resolved and an unresolved goal condition. In the unresolved goal condition, participants are instructed to identify an ongoing and unresolved concern in their goal achievement process that repeatedly comes into their mind and caused them to feel negative or stressed during the previous week. In contrast, participants in the resolved goal condition are asked to identify a concern in their goal achievement process that had previously troubled them, but that had since been resolved. Thus, the two conditions of the goal-cueing task were designed to directly contrast the impact of self-focus on resolved and unresolved goals, thereby manipulating self-discrepancy and rumination [33]. However, there are only a few studies applying this procedure and systematic evaluation is still lacking. Following the original study by Roberts and colleagues [3], Lanning [33] applied the procedure in the context of research on the influence on general memory, and Edwards [34] investigated whether an approach or avoidance framing influences rumination cued by unresolved goals. Kornacka and colleagues [28] used the unresolved condition of the goal-cueing task to activate rumination in their participants before investigating the experimental induction of abstract versus concrete repetitive negative thoughts and their influence on emotional reactivity and attentional disengagement. Roberts et al. [32] found that cueing an unresolved goal triggered more spontaneous rumination compared to cueing a resolved goal. Importantly, in the studies by Roberts et al. [3,32] and Edwards [34], state rumination was not assessed in a direct way (i.e., by means of a validated questionnaire. In contrast, state rumination was measured with probes of which one probe represented the identified concern (i.e., the resolved or unresolved problem) as index of state rumination (for more details regarding probe approach see also Kane et al. [35]). Further, Roberts et al. [3,32] (see also Edwards [34]) applied an additional outcome measure (SART: Sustained Attention to response task [36]) to test potential effects of rumination on performance in an unrelated cognitive task, assessing attentional lapses due to minimal demands on control processes. Results were mixed, as condition differences in task performance were found in one study [3]), while no differences were obtained in two other studies [32,34].

In summary, one of the advantages of the goal-cueing task is that it deals with unresolved goals in the individual goal achievement process, and because people consciously and unconsciously pursue multiple goals daily, the likelihood is very high that discrepancies will be perceived, which can lead to rumination. On the one hand, this suggests that the individual goal achievement process can be considered a reliable indicator of rumination. On the other hand, the reference to goals creates a more natural setting (compared to the response task), which produces more personally relevant reasons to ruminate [15]). Finally, referring to individual goals may increase participants’ commitment to an experiment as well as personal relevance compared to clinical settings. Overall, the goal-cueing task is promising in its application and theoretically justified, but direct evidence for inducing state rumination is lacking.

### Overview of Experiments 1a-c

The overall goal of the following three experiments is to examine the effectiveness of the goal-cueing task in different experimental settings. For this, we used different measures of state rumination covering different facets of rumination: First, we applied the BSRI, to capture the momentary occurrence of thoughts that focus one’s attention on one’s distress along with its possible causes and implications [23]. Second, we used a single rumination item according to Koval et al. [37], to measure general state rumination and third, we utilized the index of ruminative self-focus by Moberly and Watkins [14], which involves the focus on feelings as well as the focus on problems that matches discrepancy-based accounts.

Moreover, following the GPT and findings of previous studies [3,32], we focused exclusively on unresolved goals in the individual goal-achievement process to induce state rumination in participants and evaluate to what extent this condition can be modified for application in laboratory or field research. The effectivity of these modifications (i.e., shortenings of the elaborated unresolved condition to different degrees) is compared with the elaborated unresolved condition according to Roberts et al. [3] and with an additional neutral control condition in Experiments 1b-c. Specifically, we were interested in whether the use of the goal-cueing task had a direct observable effect on state rumination as assessed by different measures. In addition, we tested whether the effectivity of the goal-cueing task in inducing state rumination remains stable when the experimental condition is shortened. Therefore, in Experiment 1a, we compared the elaborated unresolved condition (i.e., 3 steps of the goal-cueing task) with an experimental condition in which step 3 (goal focus period) was omitted. Based on the results, in Experiment 1b we further shortened the experimental condition (including only step 1 of the goal-cueing task) and compared it to the elaborated unresolved condition and to a neutral control condition that was not related to personal goals. In Experiment 1c, we then reduced the goal focus period of the elaborated unresolved condition from 10 min to 5 min and compared this again with the shortened experimental condition from Experiment 1a (i.e., step 3 –goal focus period was omitted) and the neutral control condition already mentioned. We assessed further variables, using the same approach, to account for emotional processes in addition to cognitive processes (for more details see Material & Measures for each study).

Shortening the elaborated unresolved condition would have the advantage of (1) reducing the mental stress on participants, (2) allowing for application in a variety of contexts including performance contexts (in sports, in the academic or medical domain), where time economy plays an important role, and (3) future application in the field or in experimental ambulatory assessment studies.

## Experiment 1a

In the first experiment, we examined the efficacy of a shortened unresolved condition (referred to as experimental condition 2; EC2) compared to the elaborated unresolved condition (referred to as experimental condition 1; EC1) to provide information for further application. Therefore, we modified the unresolved condition so that we only applied the first two steps of the goal-cueing task (goal identification and goal evaluation) and checked whether different modifications in the unresolved condition led to same outcomes in terms of state-rumination.

### Methods

#### Participants

In our Experiment 1a, 164 participants (female: n = 101; male: n = 61) completed an online session. The mean age was 28.68 (*SD* = 13.11). Most of the participants were students with different subjects (n = 94), followed by 34 employers and 12 officers. Twenty-four of the participants had other employment relationships. Table 1 presents an overview of sample characteristics separated by condition. Moreover, participants in the two conditions did not significantly differ in their level of trait rumination, *t*(161.57) = -0.54, *p* = .59, *d* = 0.08.

**Table 1:** Mean (M), standard deviations (SD), 95% confidence intervals (95% CI) and Cronbach’s alpha (α) of sample characteristics separated by conditions for Experiments 1a-c.

		Experimental condition 1		Experimental condition 2			Neutral control condition	
	*α*	*M* (*SD*)	*95%CI*	*M* (*SD*)	*95%CI*		*M* (*SD*)	*95%CI*
***Exp*. *1a***		***n* = 87** (f = 51, m = 36)		***n* = 77** (f = 52, m = 25)				
Age		29.82 (13.13)	-	27.41 (13.06)		-		
PTQ	.92	24.82 (9.17)	[22.73, 26.90]	24.01 (9.83)		[21.91, 26.11]		
RSQ-B	.72	9.23 (2.83)	[8.59, 9.88]	8.77 (2.80)		[8.17, 9.37]		
RSQ-R	.70	10.17 (2.74)	[9.54, 10.79]	9.50 (3.12)		[8.84, 10.17]		
GSE	.85	30.55 (4.14)	[29.67, 31.43]	29.97 (4.00)		[29.07, 30.88]		
***Exp*. *1b***		**n = 20** (f = 15, m = 5)		**n = 9** (f = 5, m = 4)			**n = 10** (f = 7, m = 3)	
Age		26.70 (5.99)	-	27.44 (10.12)		-	24.10 (4.58)	-
PTQ	.96	21.00 (10.77)	[15.96, 26.04]	20.22 (16.74)		[7.35, 33.09]	20.00 (13.37)	[10.44, 29.56]
RSQ-B	.71	7.85 (1.87)	[6.97, 8.72]	9.44 (3.81)		[6.51, 12.37]	9.10 (3.03)	[6.93, 11.27]
RSQ-R	.68	10.25 (3.27)	[8.72, 11.78]	10.67 (3.24)		[8.17, 13.16]	9.50 (3.41)	[7.06, 11.94]
GSE	.87	31.15 (4.94)	[28.83, 33.46]	30.50 (3.94)		[27.52, 33.58]	30.80 (4.37)	[27.68, 33.92]
**Exp. 1c**		**n = 20** (f = 14, m = 6)		**n = 19** (f = 13, m = 6)			**n = 20** (f = 14, m = 6)	
Age		24.35 (7.52)		25.37 (4.47)			24.60 (3.47)	
PTQ	.94	25.40 (10.96)	[20.27, 30.53]	26.31(11.61)		[20.72, 31.91]	26.10 (10.59)	[21.14, 31.05]
RSQ-B	.77	9.70 (2.99)	[8.30, 11.10]	9.42 (3.02)		[7.96, 10.88]	9.60 (2.64)	[8.36, 10.83]
RSQ-R	.67	10.40 (3.22)	[8.89, 11.91]	10.00 (2.31)		[8.89, 11.11]	9.55 (3.00)	[8.15, 10.95]
GSE	.83	29.10 (4.13)	[27.17, 31.03]	28.47 (3.73)		[26.67, 30.27]	29.00 (3.85)	[27.20, 30.80]

*Note*. Exp. = experiment, Experimental condition 1 = elaborated unresolved condition, Experimental condition 2 = shortened unresolved condition (omitted goal-focus period), n = number of participants, f = female, m = male, PTQ = Perseverative Thinking Questionnaire, RSQ-B = Brooding subscale of Response Styles Questionnaire, RSQ-R = Reflection subscale of the Response Styles Questionnaire, GSE = General Self-Efficacy Scale.

#### Procedure

Participants were invited to participate in the experiments via institutes’ student mailing list, student contacts, and notices on the bulletin board. The experiment consisted of two parts that both were completed online at home due to the ongoing pandemic at the time of experiment. First, participants completed an initial online survey and then completed the experimental part second.

The initial survey was conducted using *SoSci Survey* [38] and comprised biographical and sociodemographic questions as well as different personality questionnaires (see below for detailed descriptions of the utilized questionnaires). The questionnaire data was also the basis for a stratified randomization of the participants. In detail, we first grouped participants according to their level of trait rumination (measured with the Perseverative Thinking Questionnaire; PTQ, [27]) and their gender, and second randomly assigned them to one of the two experimental conditions (or to a neutral control condition in Experiment 1b and 1c). We thus ensured that the participants in the different conditions did not differ significantly from each other in these characteristics at the group level.

The study protocol was approved by the local Review Board of the Institute for Psychology of the Johannes Gutenberg-University Mainz and was conducted according to the guidelines of the Declaration of Helsinki. Participants were informed about the nature and the procedure of the experiment and gave consent before completing the questionnaires. Participation in this experiment was voluntary. Psychology students received course credits for participating. This procedure also corresponds to that of the following two experiments, unless otherwise stated.

#### Advantage and disadvantage of applying an online experiment in our study

On the one hand, an online experiment has some advantages, such as facilitation regarding sample size, flexibility of data collection, and time economy. On the other hand, the main disadvantages of online experiments include the difficulty to control the situation in which participants complete the task and thus the standardization of data collection, as well as the more difficult protection of the participants from stress exposure [39]. Therefore, we took a few steps to increase the quality of the implementation of the online experiments and to ensure the protection of participants from negative emotional reactions or stress.

First, even though in previous experiments in our department, there were no negative emotional reactions of the participants after the experimental setting, potential negative reactions cannot be detected in the online experiments due to the lack of interaction. Consequently, before participants had the possibility to complete the initial survey, we conducted a screening in advance to exclude vulnerable individuals from the experiment. Exclusion criteria were an experienced traumatic event in the past, an actual diagnosed mental disorder, or a current psychotherapeutic treatment. In addition, we provided our contact details on several occasions, enabling participants to contact us if they felt unwell or found themselves in a bad emotional state.

Second, to increase the understanding of important information and the procedure of the experiment, as well as to reduce the number of confounding variables, participants received detailed instructions on how to conduct the experiment prior to their participation. We instructed the participants to find a quiet place for the duration of the experiment where they would be undisturbed and not distracted by friends or family, the radio, television, or their smartphone. Participants were advised to switch off their smartphone or put it in airplane mode and find a place where they could be themselves and did not have to leave for 60 minutes.

Third, to get an idea about the quality of performance in the online experiment, we asked participants at the end of the experiment (1) how focused they were, (2) how seriously they tried to implement the instructions on how to perform the experiment, and (3) how well they succeeded. Participants answered these three questions on a 5-point scale ranging from ‘1’ (*not at all*) to ‘5’ (*very*). Participants indicated that their concentration was 3.79 (*SD* = 1.00), on average they tried to follow the instructions seriously with 4.51 (*SD* = 0.70), and on average they succeeded with a mean of 4.06 (*SD* = 0.93) on the 5-point scale. Furthermore, participants in the two conditions showed no significant differences in the three quality measures (all *p*_wilcox_‘s > .05).

#### Measures & material

We used the German versions of the following questionnaires. Unless otherwise stated, these questionnaires were used in all four experiments. Means, standard deviations, 95% confidence intervals as well as Cronbach’s alpha for the present sample are summarized in Table 1.

*Perseverative thinking*. The Perseverative Thinking Questionnaire (PTQ; [27]) is a content-independent self-report questionnaire of repetitive negative thoughts. The PTQ consists of 15 items (e.g., “Thoughts come to my mind without me wanting them to”) and is rated on a 5-point scale, ranging from ‘0’ (*never*) to ‘4’ (*almost always*). Here, we report the general PTQ score. Cronbach’s alpha for the entire PTQ is α = 0.95 for the original study [27].

*Brooding and reflection*. Huffziger and Kühner [40] validated the 10-item short version of the Response Styles Questionnaire (RSQ; original English version: Treynor et al. [41]; long version: Nolen-Hoeksema [42]) with the facets Reflection and Brooding. In the RSQ, it is assumed that brooding describes dysfunctional ruminating about an unattained goal (e.g., “What am I doing to deserve this?"), while reflection describes a more goal- and solution-oriented self-reflection (e.g., “I write down what I am thinking and analyze it.”). Each scale comprises five items. Participants rated all 10 items on a 4-point Likert Scale ranging from ‘1’ (*almost never*) to ‘4’ (*almost always*). Cronbach’s α for the original study is .60 for brooding and .73 for reflection [40].

*Self-efficacy*. We used the General Self-Efficacy Scale (GSE; Hinz et al. [43]; English version: Schwarzer & Jerusalem [44]) to assess participants’ general sense of perceived self-efficacy, for instance in relation to coping with everyday life or after experiencing all kinds of stressful life events. The GSE comprises 10 items (e.g., “I can always manage to solve difficult problems if I try hard enough.”) and is answered on a 4-point scale (1 = *not at all true*, 2 = *hardly true*, 3 = *moderately true*, 4 = *exactly true*). Cronbach’s α in the original study is .92 [43].

*State rumination*. We tested state rumination with two measures: First, we assessed the 8-item Brief State Rumination Inventory (BSRI, Marchetti et al. [23] to capture the current level of repetitive negative thinking at the time of answering. The BSRI was designed to capture maladaptive state rumination defined as “the momentary occurrence of thoughts that focus one’s attention on one’s distress along with its possible causes and implications ([23], p.2). All eight statements of the BSRI (e.g., “Right now, it is hard for me to shut off negative thoughts about myself”) were answered on an 11-point scale ranging from ‘0’ (*not at all*) to ‘10’ (*very*). We have translated the items into German for the purpose of our experiments. However, the items in the translated version are not validated. Cronbach’s α of the original study is .89 and .91 for before and after an experimental manipulation [23]. Second, we used a single item (“To which extent did you ruminate over something?”, see also Koval et al. [37]) to assess general state rumination (hereinafter referred to as general rumination rating), which was also rated on an 11-point scale ranging from ‘0’ (*not at all*) to ‘10’ (*very*).

*Perceived strain*. In addition, participants complete the same 11-point scale on how much they felt burdened by the problem.

It should be noted that there is a risk that the use of single-item scales may not adequately capture the construct. Nevertheless, the use of such single-item scales is also recommended [45] because they are “easier and take less time to complete, may be less expensive, may contain more face validity, and may be more flexible than multiple-item scales” [46, p. 77].

*Mood*. We assessed the Multidimensional Mood Questionnaire (MDMQ; Wilhelm & Schoebi [47]) to measure three basic dimensions of mood–valence, energetic arousal, and calmness. The MDMQ consists of six items and is a bipolar measure that comprises three pairs of adjectives rated on a 7-point scale describing opposite end points of different mood dimensions (e.g., energetic arousal: tired vs. awake, full of energy vs. without energy). Cronbach’s α of the three scales ranged from .73 to .89 in the original study [47].

*Goal-cueing task.* The goal-cueing task by Roberts et al. [3] can be divided into three steps, i.e., identification, evaluation and focusing period of a personal relevant goal. In the EC1 (i.e., unresolved goal condition), participants are instructed to identify an ongoing unresolved goal that repeatedly troubled them, causing them to feel sad, negative, or stressed during the previous week. Participants are also provided with appropriate examples of problems. Then, participants briefly outline their problem in 5 to 10 sentences. In step 2 (evaluation), participants indicate the extent to which the unresolved hamper their individual goal achievement process at two points in time, the present time, and the time when it was at its worst. Further, participants indicate how important the goal is, how much the problem in the individual goal achievement process exemplifies more general problems, how long the problem exists and how much time they spend thinking about the problem last week. Step 3 consists of a 10-minute goal-focused period, where participants work through a pre-recoded script delivered over headphones, which guide them thorough focusing on the identified unresolved concern. Example instructions are “Think about what is important about this difficulty in terms of your personal goals”, or “Focus on the aspects of the difficulty that repeatedly come to mind”. Based on findings of Klinger et al. [48, p. 3] showing that cues may take many forms, for example, “a word, an image, or a smell that is associated with an ongoing goal pursuit (including cues related to failure to achieve a goal)”, we extended the original goal-focusing period to increase the induction of state rumination. We then added seven new instructions at the beginning of the goal focus period that trigger all five senses of the participants (e.g., “What did the situation look like in concrete terms?… What did you see? Remember all the sounds you perceived in the situation?…Try to remember every detail of your surroundings.”) to increase the probability of finding a trigger for spontaneous state rumination. We also shortened the intervals between the instructions so that the total time of 10 minutes was not exceeded.

**Fig 1:**
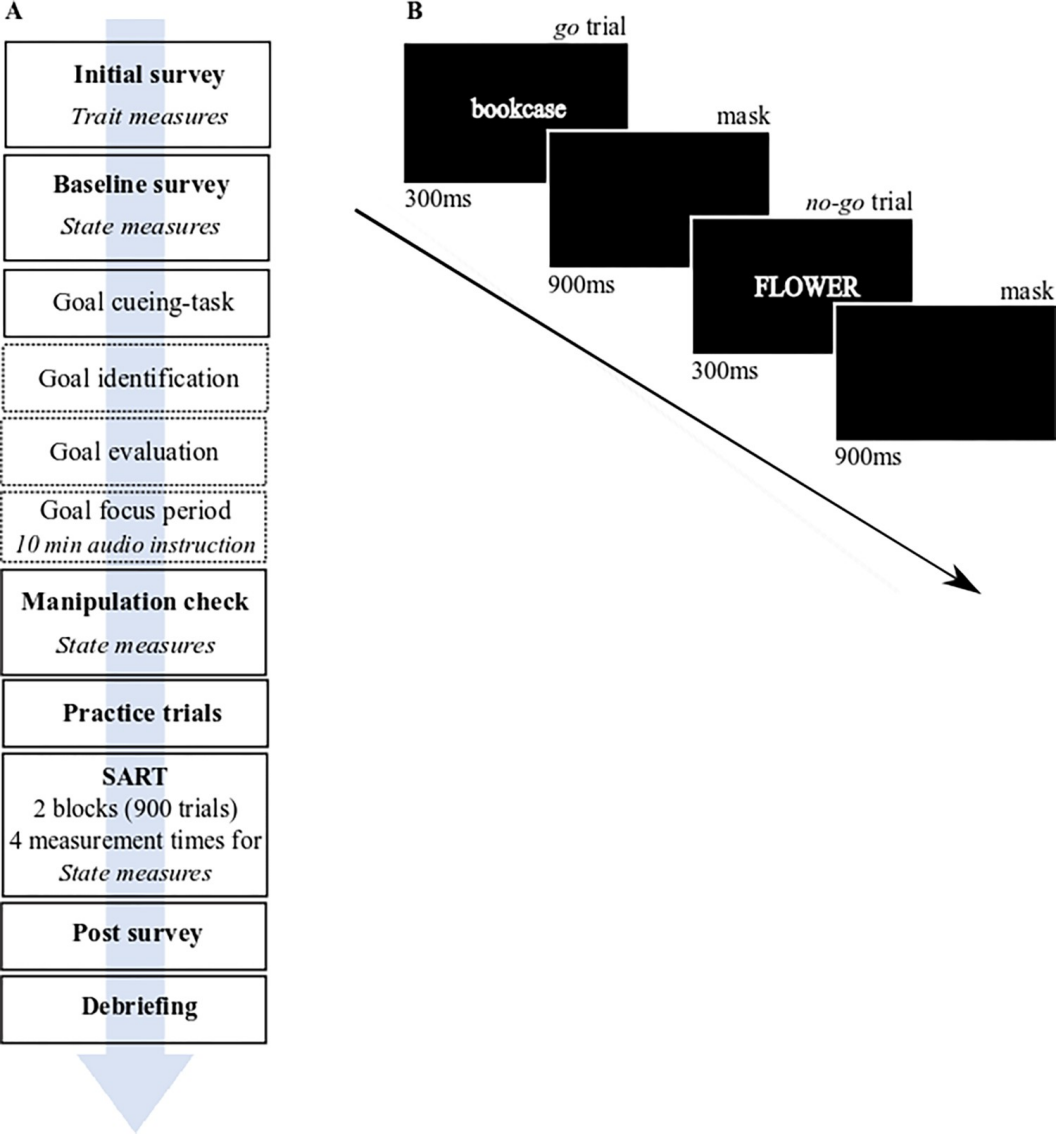
(A) Overview of the procedure of Experiment 1a. After participants completed the initial survey, they were assigned to the different conditions and given access information for the experimental session. The experimental session started with a baseline survey, in which the current levels of state rumination, mood, and perceived strain were collected. Subsequently, the goal-cueing task, with its three steps–step1: Goal identification, step 2: Goal evaluation and, step 3: Goal focus period) was carried out. The manipulation check, which included the same items as the baseline survey followed. To get familiar with the task, participants played 18 practice trials before they started with the first bock of the SART (for Experiments 1c and 2, the practice trials were conducted at the beginning of the experimental session before the baseline survey). Participants played two blocks with self-determined breaks, in which the current level of state rumination, mood, and perceived strain were collected. We also performed the same query after 60% of no-go trials in every block and after the SART ended. In addition, five post questions concerning the current evaluation of the identified goal followed. Finally, the debriefing took place in which the participants are fully informed about the objectives of the experiment. (B) shows a sequence of the SART, consisting of a go-trial (lowercase word) and a no-go trial (uppercase word), which are displayed 300ms each. In between appeared a mask for 900ms.

*Sustained Attention to Response Task (SART)*. The SART [36] is a simple go/no-go paradigm in which neutral words (e.g., “father”, “shirt”, or “green”) are presented in white text on a black background in the center of the screen. Each word appeared individually on screen for 300ms followed by a 900ms mask (see Fig 1). The participants’ task was to respond to words in lowercase letters as quickly as possible by pressing the space bar (go-trials, e.g., “flower”). When a word appeared in upper case, participants were required to withhold their response (no-go-trial, e.g., “CHURCH”). The SART comprises in its shortened version two blocks of 450 trials each, consisting of 45 words repeated ten times in a different order. Within each set of 45 words, five uppercase words appeared randomly among 40 lowercase words. After 450 trials (i.e., one block), we allowed the participants to take a break, where they could determine the length of the break themselves up to a maximum of 3 minutes. The SART took approximately 30 minutes to complete.

*Post evaluation of the identified problem*. After completing the experimental part, participants indicated (1) how difficult it was to stop thinking about the concern, (2) to what extent their focus was mainly on negative aspects, or (3) on bad feelings, (4) to what extent thinking about the problem made it seem worse and (5) made them feel worse (questions were adapted from Mosewich et al. [49]). Participants rated all questions on a 5-point scale ranging from ‘1’ (*not at all*) to ‘5’ (*very*).

#### Data analyses

Collection of experimental data from all four experiments was carried out with the Inquisit Webplayer [50] and data preparation and all statistical analyses were performed with the software RStudio [51].

*Statistical tests*. To analyze the effects of our goal-cueing task on state-rumination, mood and perceived strain, we applied single mixed analyses of variance (ANOVAs) using the ezANOVA-function (“ez”-package; [52]) with time (pre vs. post goal-cueing task) and condition (unresolved vs. shortened unresolved [vs. control]) as a factor. Beforehand, we checked the requirements for the application (normal distribution and homogeneity of variances). We conducted a Shapiro-Wilk Test for testing the assumption of normality (*p* > .05) and a Levene’s Test for testing the homogeneity of variance (*p* > .05). Given the data we collected to test the effectiveness of the experimental setting in different contexts, we were also able to conduct an exploratory analysis related to gender differences to provide further information to researchers and practitioners. For this purpose, we included gender as an additional factor in our ANOVA. Since this analysis was not the focus of our research question, we did not formulate a specific hypothesis in this regard. However, previous studies highlighted the tendency of women to show more rumination as opposed to men [41,53,54]. The results of the exploratory analyses are summarized in S1 Table.

We additionally applied single mixed ANOVAs with time (four time points during the SART) and condition (unresolved vs. modified unresolved goal) as factors to investigate whether the goal-cueing task also led to changes of state rumination, mood, and perceived strain during the SART. Before that, we checked whether the data fulfilled the requirements. In case of violation of sphericity, we used Mauchly’s Test (*p* < .05) and applied a Greenhouse-Geisser correction for ANOVAs. Since these analyses were not the focus of our experiments, but we are pleased to provide the information for further research purposes, we report the results in S2 and S3 Tables.

To compare the SART task performance across participants in different conditions, we examined the errors of commission (i.e., incorrect responses: key presses in no-go trials) as well as the mean reaction times (mean RTs) for correct go-trials. According to Cheyne et al. [55], mean RTs were calculated for all response latencies over 200ms. Reaction times less than 100ms were coded as anticipatory and reaction times between 100ms and 200ms were coded as ambiguous. We then applied mixed ANOVAs with time (SART: Block 1 vs. Block 2) and condition (e.g., unresolved vs. shortened unresolved [vs. control]) as factors.

Regarding participants’ characterization of problems in the individual goal achievement process and their evaluation of the identified problem after the experimental setting, we analyzed mean differences between conditions with independent *t*-tests. In case of non-parametric distribution, we reported the significance of Wilcoxon signed-rank test as robust alternative for independent *t*-tests (*p*_*wilcox*_; Field et al. [56]).

*Effect sizes*. We report the effect size of mean differences between conditions with Cohen’s d [57] with the following criteria: *d* = 0.20, *d* = 0.50 and *d* = 0.80 for small, medium, and large effects (see also Fritz et al. [58] for interpretation). In case of non-parametric distribution, we report the significance of Wilcoxon signed-rank test with corresponding robust effect size (r). The interpretation values for *r* are: 0.10 to < 0.30 for a small effect, 0.30 to < 0.50 for a moderate effect and ≥ 0.50 for a large effect [58]. For the ANOVAs, we report partial eta squared (ηp2) as a measure of effect with the following criteria for small, medium, and large effect: 0.01, 0.06, and > 0.14 [59,60].

### Results

#### Characterization of problems in the individual goal achievement process

Considering the results of the characterization of the identified problems, there were no differences between the participants in both conditions. That is, there were no differences in terms of how the unresolved goal hampered participants individual goal achievement process at the present time and at the time when it was at its worst, how important the goal was, how much the problem in the individual goal achievement process exemplified more general problems, how long the problem existed and how much time they spent thinking about the problem last week. The upper part of Table 2 summarizes the descriptive statistics and the results of the independent *t*-tests for participants’ goal-evaluations for Experiments 1a-c.

**Table 2:** Mean (M), standard deviations (SD), test statistics, and effect sizes of participants goal characteristics and goal evaluations after the experiment for Experiments 1a-c, each separated by condition.

	EC1		EC2			
*Goal characteristics*	*M*	*SD*	*M*	*SD*	Test statistic	Effect size
***Exp*. *1a***						
(1) Trouble at the time of the experiment	7.40	2.00	7.02	2.49	*p*_*wilcox*_ = .52	*r* = .11
(2) Trouble in its worst…	8.99	1.08	8.61	2.02	*p*_*wilcox*_ = .98	*r* < .01
(3) Goal Importance	8.22	1.77	8.25	2.29	*p*_*wilcox*_ = .36	*r* = .07
(4) Example for general problems	4.46	2.91	4.40	2.96	*p*_*wilcox*_ = .90	*r* < .01
(5) Duration of the problem	5.53	2.83	6.06	2.89	*p*_*wilcox*_ = .24	*r* = .09
(6) Time spent with the problem	6.63	2.38	6.10	2.44	*p*_*wilcox*_ = .14	*r* = .11
***Exp*. *1b***						
(1) Trouble at the time of the experiment	7.30	2.68				
(2) Trouble in its worst…	8.65	2.37				
(3) Goal Importance	7.95	2.74				
(4) Example for general problems	5.10	3.37				
(5) Duration of the problem	5.75	2.63				
(6) Time spent with the problem	6.15	2.89				
***Exp*. *1c***						
(1) Trouble at the time of the experiment	7.15	1.98	7.47	2.27	*p*_*wilcox*_ = .46	*r* = .12
(2) Trouble in its worst…	9.10	1.80	9.05	1.27	*p*_*wilcox*_ = .46	*r* = .12
(3) Goal Importance	8.40	1.70	8.47	1.61	*p*_*wilcox*_ = .99	*r* < .01
(4) Example for general problems	5.20	3.35	5.47	2.54	*t*(35.34) = - 0.29, *p* = .77	*d* = 0.09
(5) Duration of the problem	6.15	3.01	6.31	2.54	*p*_*wilcox*_ = .83	*r* = .03
(6) Time spent with the problem	6.05	2.62	7.42	2.34	*t(36*.*86) = -1*.*72*, *p =* .*09*	*d* = 0.55
***Goal evaluation*** ***Exp*. *1a***						
(1) Difficulties stop thinking about the problem	2.38	1.10	1.88	0.98	*p*_*wilcox*_ < .01	*r* = .24
(2) Problem seemed worse	2.27	1.30	2.12	1.13	*p*_*wilcox*_ = .52	*r* = .05
(3) Participant felt worse	2.52	1.34	2.43	1.24	*p*_*wilcox*_ = .75	*r* = .02
(4) Focus on negative aspects	2.90	1.18	2.74	1.16	*p*_*wilcox*_ = .42	*r* = .06
(5) Focus on bad feelings	2.57	1.12	2.38	1.12	*p*_*wilcox*_ = .27	*r* = .09
***Exp*. *1b***						
(1) Difficulties stop thinking about the problem	1.95	1.10	2.33	1.12	*p*_*wilcox*_ = .33	*r* = .18
(2) Problem seemed worse	2.45	1.23	2.33	1.22	*p*_*wilcox*_ = .83	*r* = .04
(3) Participant felt worse	2.95	1.28	2.89	1.05	*p*_*wilcox*_ = .77	*r* = .05
(4) Focus on negative aspects	2.55	1.28	2.89	0.78	*p*_*wilcox*_ = .42	*r* = .15
(5) Focus on bad feelings	2.55	1.32	2.78	1.09	*p*_*wilcox*_ = .59	*r* = .10
***Exp*. *1c***						
(1) Difficulties stop thinking about the problem	2.75	1.48	2.37	1.30	*p*_*wilcox*_ = .40	*r* = .13
(2) Problem seemed worse	2.45	1.32	2.63	1.38	*p*_*wilcox*_ = .67	*r* = .07
(3) Participant felt worse	2.80	1.32	2.89	1.37	*p*_*wilcox*_ = .89	*r* = .02
(4) Focus on negative aspects	3.00	1.21	3.10	1.28	*p*_*wilcox*_ = .77	*r* = .05
(5) Focus on bad feelings	2.30	1.08	2.63	1.46	*p*_*wilcox*_ = .61	*r* = .08

*Note*. Exp. = experiment, EC = Experimental condition; note that experimental condition 2 varies in its content in Experiment 1a to 1c, *d* = Cohens *d*, *r* = respective effect size for Wilcoxon signed-rank test.

#### Effects of goal-cueing task on state rumination, mood and perceived strain

Table 4 summarizes the results of the respective *F*-statistic for each mixed ANOVA separated by main effects.

We found no significant difference between the conditions in our experimental variables except for perceived strain, *F*(1,162) = 4.13, *p* = .04, ηp^2^ = .02, indicating higher values for participant in the EC1 compared to participants in the EC2. Furthermore, time effects were obtained in all variables used except for energetic arousal, *F*(1,162) < 1. Participants’ valence and calmness levels decreased significantly after the goal-cueing task, whereas levels of state rumination and perceived strain increased.

In addition, there was a significant interaction effect for the general rumination rating, and energetic arousal. Participants’ general state rumination was lower before than after the goal-cueing task in both experimental conditions (EC1: *p*_bonf_ < .001; EC2: *p*_bonf_ < .01) with significantly different scores between the two conditions after the goal-cueing task (*p*_bonf_ = .04). Regarding participants’ energetic arousal, post-hoc test revealed no significant differences.

#### Errors of commission and mean RT during the SART

There are no differences in SART performance between participants in either condition (see Table 5).

#### Evaluation of the identified problem/concern after the experimental setting

To retrospectively assess the impact of the identified problem on various aspects and to examine respective potential differences between conditions, participants answered five different questions. Results indicated that participants in the EC1 had significantly more difficulty stopping to think about the problem than participants in the EC2 (*p*_*wilcox*_ < .01, *r* = .24). There were no differences in the other goal evaluation questions. Table 2 summarizes mean values and standard deviations, as well as the respective test statistic for each experiment.

### Summary of the results

The results confirm a successful induction of state rumination in that values of both state rumination measures increase from before to after the goal-cueing task. Interestingly, an additional interaction effect shows that the values after the goal-cueing task are significantly higher in the EC1 than in EC2. However, this applies only for the general rumination rating and the effect can be considered small. In terms of mood, there was essentially a decrease over time (except for energetic arousal) and perceived strain showed an inverse effect. In addition, there were no differences in performance on the SART and no differences in problem characterization. Furthermore, regarding post-evaluation, participants of EC1 reported more difficulties to stop thinking about the problem (which is a definitional component of rumination) than participants of EC2.

### Discussion Experiment 1a

Shortening the instructions of the unresolved condition (i.e., omitting step 3 –goal focus period) did not in principle affect the results and led to similar outcomes in terms of state-rumination on both measures compared to the elaborated unresolved condition. This suggests that both experimental conditions can be used equally to induce state rumination in the participants. However, the experiment is limited by the absence of a control condition. To make a valid statement regarding the effectivity of the two experimental conditions, they must be compared with a control condition in a next step.

In general, the shortened version would have the advantage of saving time and reducing mental stress for the participants. This would be beneficial for future application in laboratory and in field studies. To this end, it would be necessary to verify whether the difference between the two ECs in terms of participants’ perceived strain disappears because it is due to the different duration of the conditions (and thus to a difference in intensity), or whether other causes underlie the difference in strain perception.

## Experiment 1b

Results from Experiment 1a suggested that the identification of a problem in the individual goal achievement and its evaluation is sufficient to produce state rumination. The aim of the second experiment was now to compare the experimental condition (i.e., the elaborated unresolved condition) with a further shortened experimental condition (i.e., omitting step 2 –goal evaluation and step 3 –goal focus period) to examine whether the effects remain stable with respect to state rumination. To better interpret potential effects, we also added a neutral control condition [i.e., NCC], which does not refer to individual goals.

### Method

#### Participants

Overall, forty psychology students were recruited from the Johannes Gutenberg-University Mainz for the online experiment. However, we excluded one participant who reported not making a serious effort to follow the implementation instructions from further data analysis (see also *Evaluation of the quality of the online experiment*). Thus, 39 participants (female: n = 27; male: n = 12) were included in the following analysis. At the time of the data collection, 20 students were enrolled in the bachelor and 19 in the master program. The mean age was 26.20 (*SD* = 6.79), and participants received course credits for participating. The lower part of Table 1 presents an overview of sample characteristics separated by condition. Moreover, participants in the three conditions did not significantly differ in their level of trait rumination, *F*(2,36) = 0.02, *p* = .97, ηp^2^ < .01.

#### Evaluation of the quality of the online experiment

Participants rated the quality of their performance, indicating that their concentration was 3.67 (*SD* = 0.84), that they made an average effort of 4.54 (*SD* = 0.68) to follow the instructions seriously, and that they succeeded with 4.05 (*SD* = 1.00). Furthermore, participants in the three conditions did not differ significantly on these three quality measures (all *F*’s [2,36] < 1).

#### Measures & material

*Goal-cueing task*. The EC1 remained the same as in the previous experiment. EC2 was a shortened version of the original unresolved condition, in which participants identified only a problem in their individual goal achievement process (step 1), whereas participants in the NCC were given a neutral writing task that did not relate to personal goal but had the same extent. Participants were asked to describe what they did from the morning until the time of the experiment [61,62] instead of identifying a problem of their individual goal achievement process.

### Results

#### Characterization of problems in the individual goal achievement process

The characterization of the identified problem refers solely to EC1, since this part was not included in EC2. Table 2 shows mean values and standard deviations for comparison with the other experiments.

#### Effects of goal-cueing task on state rumination, mood, and perceived strain

Mean values and standard deviations of the relevant experimental variables are presented in Table 3 and the results of the respective *F*-statistic for each mixed ANOVA are summarized in Table 4.

**Table 3:** Mean values and standard deviations of relevant experimental variables separated by condition and time for Experiments 1a-c.

		Experimental condition 1		Experimental condition 2		Neutral control condition	
		Pre	Post	Pre	Post	Pre	Post
***Exp*. *1a***	***State Rumination***						
	BSRI	2.60 (2.00)	3.44 (2.22)	2.62 (1.94)	3.16 (2.24)		
	General rumination rating	3.92 (2.94)	6.70 (2.60)	3.78 (2.67)	5.51 (3.01)		
	***Mood***						
	Energetic Arousal	3.56 (1.07)	3.35 (1.12)	3.44 (1.16)	3.56 (1.28)		
	Valence	4.06 (1.11)	3.34 (1.28)	4.17 (1.23)	3.77 (1.28)		
	Calmness	4.14 (1.27)	3.70 (1.41)	4.57 (1.17)	3.69 (1.27)		
	***Perceived strain***	2.72 (2.90)	5.49 (2.89)	2.38 (2.57)	4.36 (2.81)		
***Exp*. *1b***	***State Rumination***						
	BSRI	2.99 (1.85)	4.01 (2.11)	3.17 (2.16)	4.39 (2.74)	1.82 (1.89)	1.70 (2.32)
	General rumination rating	5.30 (2.77)	6.90 (2.05)	3.22 (2.73)	6.67 (1.94)	3.00 (2.40)	2.20 (1.81)
	***Mood***						
	Energetic Arousal	3.60 (1.27)	2.62 (1.11)	3.50 (0.93)	3.61 (1.02)	3.05 (1.23)	3.50 (1.25)
	Valence	4.15 (0.96)	3.00 (0.93)	3.94 (1.38)	3.11 (1.58)	3.50 (0.88)	4.55 (1.09)
	Calmness	3.77 (1.27)	3.60 (1.22)	4.28(1.00)	3.28 (1.54)	4.45 (0.86)	4.80 (1.03)
	***Perceived strain***	4.00 (2.81)	6.60 (2.04)	2.67 (2.64)	6.00 (2.45)	1.40 (0.97)	0.90 (1.59)
***Exp*. *1c***	***State Rumination***						
	BSRI	2.96 (1.98)	4.14 (2.21)	2.49 (1.85)	3.59 (2.48)	2.87 (1.84)	1.97 (1,50)
	Ruminative self-focus	3.12 (1.69)	5.32 (1.44)	3.60 (1.45)	4.95 (1.39)	3.65 (1.56)	3.10 (1.16)
	***Mood***						
	Energetic Arousal	3.20 (1.23)	3.05 (1.33)	3.21 (0.96)	3.29 (1.15)	3.17 (1.51)	3.50 (1.38)
	Valence	3.80 (1.39)	3.47 (1.26)	3.39 (1.05)	3.47 (1.27)	4.00 (1.37)	4.22 (1.55)
	Calmness	3.80 (1.08)	3.77 (1.51)	3.71 (1.36)	3.76 (1.61)	3.95 (1.44)	3.97 (1.30)
	***Perceived strain***	2.35 (1.31)	4.60 (1.67)	2.53 (1.43)	4.53 (1.68)	2.80 (1.88)	2.30 (1.69)

*Note*. Exp. = experiment, pre = measurement before the goal-cueing task, post = measurement after the goal-cueing task, BSRI = Brief State Rumination Inventory.

**Table 4:** Test statistics for pre-post comparisons of the goal-cueing task and corresponding effect sizes (ηp^2^) of the respective mixed ANOVAs separated by effects for Experiments 1a-c.

	Condition effect		Time effect		Interaction effect	
	Results *F*-statistic	ηp^2^	Results *F*-statistic	ηp^2^	Results *F*-statistic	ηp^2^
***Exp*. *1a***						
***State Rumination***						
BSRI	*F*(1,162) < 1	-	*F*(1,162) = 33.99, *p* < .001	.17	*F*(1,162) = 1.63, *p* = .20	.01
General rumination rating	*F*(1,162) = 3.37, *p* = .07	.02	*F*(1,162) = 83.04, *p* < .001	.34	*F*(1,162) = 4.54, *p* = .03	.03
***Mood***						
Energetic Arousal	*F*(1,162) < 1	-	*F*(1,162) < 1	-	*F*(1,162) = 5.49, *p* = .02	.03
Valence	*F*(1,162) = 2.77, *p* = .10	.02	*F*(1,162) = 29.87, *p* < .001	.16	*F*(1,162) = 2.37, *p* = .12	.01
Calmness	*F*(1,162) < 1		*F*(1,162) = 19.45, *p* < .001	.11	*F*(1,162) < 1	-
***Perceived strain***	*F*(1,162) = 4.13, *p* = .04	.02	*F*(1,162) = 93.69, *p* < .001	.37	*F*(1,162) = 2.54, *p* = .11	.01
***Exp*. *1b***						
***State Rumination***						
BSRI	*F*(2,36) = 3.28, *p* = .05	.15	*F*(1,36) = 6.08, *p* = .02	.14	*F*(2,36) = 1.97, *p* = .15	.10
General rumination rating	*F*(2,36) = 15.24, *p* < .001	.46	*F*(1,36) = 6.08, *p* = .02	.14	*F*(2,36) = 3.82, *p* = .03	.17
***Mood***						
Energetic Arousal	*F*(2,36) < 1	-	*F*(1,36) < 1		*F*(2,36) = 5.88, *p* < .01	.25
Valence	*F*(2,36) < 1	-	*F*(1,36) = 2.94, *p* = .09	.07	*F*(2,36) = 14.77, *p* < .001	45
Calmness	*F*(2,36) = 3.26, *p* = .05	.15	*F*(1,36) = 1.38, *p* = .25	.04	*F*(2,36) = 2.31, *p* = .11	.11
***Perceived strain***	*F*(2,36) = 19.38, *p* < .001	.52	*F*(1,36) = 13.69, *p* < .001	.27	*F*(2,36) = 5.19, *p* = .01	.22
***Exp*. *1c***						
***State Rumination***						
BSRI	*F*(2,56) = 1.82, *p* = .17	.06	*F*(1,56) = 6.66, *p* = .01	.11	*F*(2,56) = 14.94, *p* < .001	.35
Ruminative self-focus	*F*(2,56) = 3.55, *p* = .03	.11	*F*(1,56) = 20.83, *p* < .001	.27	*F*(2,56) = 14.04, *p* < .001	.33
***Mood***						
Energetic Arousal	*F*(2,56) < 1	-	*F*(2,56) < 1	-	*F*(2,56) = 1.92, *p* = .15	.06
Valence	*F*(2,56) = 1.60, *p* = .21	.05	*F*(2,56) < 1	-	*F*(2,56) = 1.46, *p* = .24	.05
Calmness	*F*(2,56) < 1	-	*F*(2,56) < 1	-	*F*(2,56) < 1	-
***Perceived strain***	*F*(2,56) = 2.99, *p* = .06	.09	*F*(1,56) = 36.66, *p* < .001	.39	*F*(2,56) = 18.28, *p* < .001	.39

*Note*. Exp. = Experiment, BSRI = Brief State Rumination Inventory.

*State rumination*. With respect to the state ruminations measures, a significant interaction effect for the general ruminations rating was observed in addition to significant time and condition effects. Post-hoc tests indicated a significant difference before and after the goal-cueing task for EC2 (*p*_*bonf*_ = .04). Furthermore, both experimental conditions differ significantly in their general state rumination after the goal-cueing task from the NCC (EC1: *p*_*bonf*_ < .001; EC2: *p*_*bonf*_ = .001). Finally, we found no differences between the two experimental conditions after the goal-cueing task and no differences before the goal-cueing task for all conditions.

*Perceived strain*. A similar pattern of results emerged for the perceived strain, where participants in NCC reported significant lower perceived strain after the goal-cueing task than the EC1 and the EC2 (both ECs: *p*_*bonf*_ *<* .001). Differences in perceived strain before and after the goal-cueing task were evident only for both ECs (EC1: *p*_*bonf*_ *<* .01; EC2: *p*_*bonf*_ *=* .04). There were no significant differences between conditions before the goal-cueing task.

*Mood*. For mood, there were no significant main effects for either condition or time. However, significant interactions were found for energetic arousal and valence, although these related only to differences in valence before and after the goal-cueing task in the EC1 (*p*_*bonf*_ *=* .02) and to differences after the goal-cueing task between EC1 and NCC (*p*_*bonf*_ *<* .01).

#### Errors of commission and mean RT during the SART

With regard to participants SART performance, results revealed a single significant effect for the factor time, *F*(1,36) = 5.70, *p* = .02, ηp^2^ = .14, indicating that more errors of commission were made as time went on (see Table 5).

**Table 5:** Mean values (M) and standard deviations (SD) of errors of commission (in percent) and mean RTs for correct go-trials during the Sustained Attention to Response task (SART) for Experiments 1a-c.

	Block 1	Block 2	Results *F*-statistic	
*Exp*. 1a*	*M (SD)*	*M (SD)*	Errors of commission	Mean RT
***Experimental condition 1***			*F*_C_(1,156) < 1	*F*_C_(1,156) < 1
Errors of commission	37.01(20.62)	39.98 (24.40)	*F*_M_(1,156) < 1	*F*_M_(1,156) = 1.58, *p* = .21, ηp^2^ = .01
Mean RT	419.15 (84.33)	417.66 (86.63)	*F*_I_(1,162) = 2.42, *p* = .12, ηp^2^ = .01	*F*_I_ (1,156) = 2.54, *p* = .11, ηp^2^ = .02
***Experimental condition 2***				
Errors of commission	41.29 (19.85)	40.46 (23.51)		
Mean RT	403.96 (79.27)	416.62 (93.00)		
***Exp*. *1b***				
***Experimental condition 1***			*F*_C_(2,36) < 1	*F*_C_(2,36) < 1
Errors of commission	38.40 (18.96)	40.10 (22.72)	*F*_T_(1,36) = 5.70, *p* = .02, ηp^2^ = .14	*F*_T_(1,36) < 1
Mean RT	418.18 (83.89)	431.92 (88.18)	*F*_I_(2,36) = 1.19, *p* = .31, ηp^2^ = .06	*F*_I_(2,36) = 2.54, *p* = .09, ηp^2^ = .12
***Experimental condition 2***				
Errors of commission	37.55 (25.82)	41.78 (29.69)		
Mean RT	457.81 (83.82)	450.33 (78.86)		
***Neutral control condition***				
Errors of commission	36.00 (18.93)	45.00 (21.44)		
Mean RT	426.93 (129.35)	394.32 (97.62)		
*Exp*. 1c*				
***Experimental condition 1***				
Errors of commission	47.68 (22.02)	48.21 (22.24)	*F*_C_(2,50) = 1.93, *p* = .15, ηp^2^ = .07 *F*_T_*(1*,*50) = 4*.*41*, *p =* .*04*, ηp^2^ = .08 *F*_I_(2,50) < 1	*F*_C_(2,50) < 1 *F*_T_(1,50) = 2.86, *p* = .10, ηp^2^ = .05 *F*_I_(2,50) < 1
Mean RT	387.54 (64.44)	382.34 (67.93)		
***Experimental condition 2***				
Errors of commission	34.50 (17.30)	40.12 (17.52)		
Mean RT	411.36 (57.24)	402.46 (67.18)		
***Neutral control condition***				
Errors of commission	47.33 (19.15)	52.44 (25.29)		
Mean RT	384.23 (70.84)	372.09 (70.72)		

*Note*. Exp. = experiment, Mean RT = Mean reaction time; *F*_C_ = *F*-statistic for condition effect, *F*_T_ = *F*-statistic for time effect, *F*_I_ = *F*-statistic for interaction effect. *In Experiment 1a, we excluded data from six participants and in Experiment 1c we excluded data from seven participants due to no serious reactions during the SART (valid go-trials = 0).

#### Evaluation of the identified problem/concern after the experimental setting

No differences were found between the EC1 and the EC2 regarding the evaluation of the identified problems after the experiment.

#### Summary of the results

The results of the mixed ANOVAs showed a significant interaction effect for the general rumination rating, in that both ECs were significantly different from the NCC after the goal-cueing task. Post-hoc analyses also showed a significant change in the general rumination rating from before to after the goal-cueing task for EC2. The same pattern of results applies to the participants’ perceived strain. Here, in addition to the change in EC2 over time in the goal-cueing task, an increase in perceived strain in EC1 was also noted. With respect to the BSRI, the interaction effect was again absent. In terms of mood, the only notable effect is the significant interaction between condition and time in the valence scores, which increased significantly in EC1 after the goal-cueing task and differed significantly from NCC after the goal-cueing task. The results of the SART performance analysis showed that errors of commission increased over time regardless of conditions. Finally, the goal characteristic of EC1 is comparable to the values from Experiment 1 and there are no differences between ECs in terms of the post evaluation.

### Discussion Experiment 1b

The main aim of Experiment 1b was to further shorten the experimental condition (i.e., original unresolved condition) for future application and to examine whether the effects remain stable with respect to state rumination. We also added a neutral control condition to better interpret potential effects. In detail, we maintained the EC1 from the previous experiment and compared it to two further conditions: EC2 consisted of a shortened version of the elaborated unresolved condition and included only step 1 of the goal-cueing task while NCC was not related to a personal goal. As expected, significant condition and time effects were obtained using both measures of state rumination, namely the BSRI and the general rumination rating. With respect to the latter, there was a significant condition x time interaction that, in addition to significant differences in state rumination before and after the goal-cueing task for both experimental conditions, also significantly differentiated the EC1 and EC2 from the NCC after the goal-cueing task. At this point, however, it must be noted that even though the conditions before the goal-cueing task did not differ significantly in their level of state rumination, the EC1 showed relatively high values. This could be due, among other things, to the fact that participants are already told in the instructions immediately before the experimental part that they must identify a personal problem, which could already trigger initial ruminative processes. However, the question of why the elevated values were found exclusively in the EC1 remained unanswered.

As part of the development process of the goal-cueing task, we only aimed to test whether further shortening would tend to result in comparable state rumination, so Experiment 1b is more of an exploratory approach. Further, Experiment 1b is also severely limited by the small sample size and the general homogeneous sample of psychology students. During college, reflecting on successes and failures in achieving important personal goals is a commonplace process [63]. Especially psychology students represent a target group with experience in self-regulation or at least a good knowledge of it. Thus, it cannot be ruled out that theoretical and practical knowledge of self-regulation influenced the effects of the goal-cueing task. Hence, only cautious recommendations can be made to further shorten the experimental condition for future laboratory and field applications. In addition, it must be considered that while further shortening may save time in an experimental setting, valuable information related to individual goals is lost.

## Experiment 1c

Since EC1 has so far achieved the strongest effects in Experiments 1a and 1b, but this is the "least favorable" condition due to its length and the loss of information when reducing the experimental condition to only one step of the goal-cueing task seems too large for some application contexts, we reduced the goal focus period (step 3) of the elaborated unresolved condition from 10 min to 5 min in Experiment 1c. We then compared these again with the shortened experimental condition from Study 1 (omitting step 3 –goal focus period) and the neutral control condition. In addition, we applied a new measure of state rumination, namely the ruminative self-focus index according to Moberly and Watkins [15], which captures participants’ focus on their own feelings and problems at the time of the prompt.

### Method

#### Participants

Sixty participants completed the experiment. One participant who reported not making a serious effort to follow the implementation instructions was excluded from further data analysis (see also *Evaluation of the quality of the online experiment*). Thus, 59 participants (female: n = 41, male: n = 18, *M*_*age*_ = 24.76, *SD*_age_ = 5.37) were included in the following analyses. Participants received 12 Euro as compensation for their participation in the experiment. Table 1 presents an overview of sample characteristics separated by both experimental (i.e., EC1 and EC2) and the neutral control condition (i.e., NCC). Participants in three conditions did not significantly differ in their level of trait rumination, *F*(2,56) = 0.04, *p* = .96, ηp^2^ < .01.

#### Evaluation of the quality of the online experiment

Regarding the quality of performing the online experiment, participants indicated that their concentration was 3.65 (*SD* = 0.94), that they made an average effort of 4.59 (*SD* = 0.65) to follow the instructions seriously, and that they succeeded with 4.08 (*SD* = 0.95). Participants in the three conditions did not differ significantly on the three quality measures (concentration & seriousness: *F*(2,56) < 1; success: *F*(2,56) = 1.49, *p* = .23, ηp^2^ = .05).

#### Measures & material

*Ruminative Self-focus*. In addition to the BSRI, we now measure state rumination with the momentary ruminative self-focus index from Moberly and Watkins [15], which includes two items assessing (1) to which extent participants were focused on their feelings and (2) to which extent they were focused on their problems. Both items were rated on an 11-point scale ranging from ‘0’ (*not at all*) to ‘10’ (*very*).

*Goal-cueing task*. The goal focus period of the original unresolved condition was now shortened von 10 to 5 minutes (EC1). The shortening essentially referred to the removal of the seven instructions at the beginning of the audio script that were intended to trigger the participants’ five senses. EC2 corresponds to EC2 from Experiment 1a (i.e., step3 –goal focus period was omitted, and the neutral control condition (NCC) remained the same as in Experiment 1b.

### Results

#### Characterization of problems in the individual goal achievement process

There were no differences between the two experimental conditions in terms of their characterization of the identified problem (see Table 2).

#### Effects of goal-cueing task on state rumination, mood, and perceived strain

Table 3 presents mean values and standard deviations of the relevant experimental variables. Table 4 summarizes the results of the respective *F*-statistic for each mixed ANOVA.

*Ruminative self-focus*. With respect to the ruminative self-focus of participants, a significant interaction effect was found in addition to significant time and condition effects. Post-hoc tests indicated that ruminative self-focus significantly increased from before to after the goal-cueing task (*p*_*bonf*_ < .001) in the EC1. There were no differences for the NCC (*p*_*bonf*_ > .99) and no differences for EC2 (*p*_*bonf*_ = .08). In addition, the two experimental conditions showed no significant differences in ruminative self-focus after the goal-cueing task (*p*_*bonf*_ > .99); however, both differed significantly from the NCC (EC1: *p*_*bonf*_ < .001; EC2: *p*_*bonf*_ < .01).

*BSRI*. Regarding the results of the BSRI, a significant time effect as well as a significant time x condition interaction was obtained. Subsequent post-hoc analyses showed that the NCC significantly differed in their BSRI scores from the EC1 (*p*_*bonf*_ = .01) but not from the EC2 (*p*_*bonf*_ = .18) after the goal-cueing task. Moreover, we obtained no other significant differences between conditions.

*Perceived strain*. For the main effects, the pattern of results was the same as for the BSRI. Post-hoc analysis showed that perceived strain significantly differed from before to after the goal-cueing task in the EC1 (*p*_*bonf*_ < .001) and EC2 (*p*_*bonf*_ < .01) but not in the NCC (*p*_*bonf*_ > .99). In addition, the two experimental conditions did not significantly differ in their perceived strain after the task (*p*_*bonf*_ > .99); however, both differed significantly from the NCC (EC1: *p*_*bonf*_ < .001; EC2: *p*_*bonf*_ < .001).

*Mood*. We found no significant effects for either condition or time or its interaction in the mood measures.

#### Errors of commission and mean RT during the SART

Regarding participants SART performance, results revealed a significant effect for the factor time, *F*(1,57) = 4.41, *p* = .04, ηp^2^ = .08 for errors of commission, indicating that more errors of commission were made as time went on (see Table 5).

#### Evaluation of the identified problem/concern after the experimental setting

No differences were found between the EC1 and the EC2 regarding the evaluation of the identified problems after the experiment (see Table 2).

#### Summary of the results

The results confirm a successful induction of state rumination, which is first expressed by a significant interaction with a large effect on both rumination measures. Both measures differ significantly from the NCC after the goal-cueing task, although for EC2 this only applies to ruminative self-focus. The same pattern of results was found for perceived strain. No significant effects were observed regarding mood. There were also no significant differences in terms of goal characteristic and post evaluation. Finally, the results of the SART analysis, showed a similar result as in Experiment 1b: regardless of the condition, more errors of commission were made over time.

### Discussion Experiment 1c

Since EC1 has so far produced the strongest effects in Experiments 1a and 1b but is the "least favorable" condition due to its length, the third experiment aimed to test the effectivity of the elaborated unresolved condition with a reduced goal focus period (5 min). This reduced EC1 was then compared again with EC2 from Experiment 1a (i.e., omitted step 3 –goal focus period) and NCC (i.e., not related to personal goals) from Experiment 1b. In addition, we added a new measure of state rumination, namely the rumination self-focus index according to Moberly and Watkins [15], which captures participants’ focus on their own feelings and problems.

The results underscore the findings from the previous two experiments, in the sense that now significant interaction effects were obtained for both ruminations measures, confirming the effectivity in inducing state rumination as well as the clear distinction from a neutral control condition unrelated to personal goals. With respect to SART performance, a further time effect for errors of commission was shown in addition to Experiment 1b, indicating an increase in errors of commission for all conditions. Why no other significant main effects were obtained in the SART task in Experiments 1a-c is discussed in the general discussion.

## General discussion and limitations Experiments 1a-c

In our three experiments, we focused on an experimental condition (i.e., goal-cueing task) to induce state rumination. In this task, participants were asked to identify, evaluate, and focus on a personally relevant goal that was previously unresolved and still bothering them. We further tested modifications of the task (i.e., shortenings of the elaborated unresolved condition to different degrees) and tested the effectivity of these modifications compared to the elaborated unresolved condition. The results of these three experiments demonstrate that state rumination can be induced by the application of the goal-cueing task. However, some shortcomings of the general approach of these three studies must be considered before a final conclusion can be drawn.

One limitation is that the three experiments obtained mixed results regarding the state rumination measures. While the overall pattern of results supports the assumption that the goal-cueing task can elicit rumination in an experimental context, the question remains whether all three versions are correspondingly effective. One reason for the mixed results could be that the three experiments have very different sample sizes, which leads to a potential power problem especially in Experiments 1b and 1c. To test this, we calculated post-hoc power analyses for all three experiments, which showed low power for Experiments 1a and 1b and sufficient power for Experiment 1c. In detail, results of post-hoc power analyses in terms of the strongest interaction effects were as follows: Experiment 1a: *N* = 164, **α** = .03, **η**p^2^ = .03 (*f* = .17), power = .52; Experiment 1b: *N* = 39, **α** = .03, **η**p^2^ = .17 (*f* = .45), power = .55; Experiment 1c: *N* = 59, **α** = .001, **η**p^2^ = .33 (*f* = .77), power = .91). Another drawback of our approach is the type of state rumination measures we used. To conduct our experiments, we relied on partially unvalidated measures such as our translated version of the BSRI. Although this means that the results should be interpreted with caution, this fact points to a general problem of limited measures of state rumination (especially in the German-speaking area). Complementing this, Marchetti et al. **[23]** highlighted that even available measures are often still characterized by unknown and/or unreplicated psychometric properties. To further validate the efficacy of the goal-cueing task, additional state measures should be considered that capture additional facets of rumination, such as repetitive quality, degree of abstractness, or uncontrollability of rumination processes. Thus, for future research, a 4-item measure from Rosenkranz et al. **[64]** or Krys **[18]** seems promising for capturing more facets of rumination.

Considering the results from the first three experiments (especially those from Experiment 1c), the EC1 with the reduced goal focus period (5 minutes) seems to be a reliable induction of rumination. The shortening of the goal-cueing task also has the advantage of avoiding confusion between abstract and concrete processing style of rumination. While in Experiments 1a and 1b the intention was to enhance the induction of rumination by adding items that involved the use of all senses, participants may also have been more focused on the direct, specific, and contextualized experience of an event through this procedure, which would correspond to a more adaptive, concrete processing style of rumination [1,28]. However, this is in contrast to the more abstract processing style, which refers to repetitive and difficult to control dwelling on one or more negative topics, which can be considered a more maladaptive processing style that has classically been defined as rumination [1,28]. Consequently, different processing styles and their mixture can have different effects on affect, mood, and emotion regulation [1,2,28,29,65]. Although the results of Experiments 1a and 1b pointed in the desired direction regarding state rumination, the instructions should be clearly assigned to a processing style in the future to increase the effects of the goal-cueing task. Furthermore, the use of a neutral control condition can also be seen as limiting. In Roberts’ original study [3], the control condition also related to a goal. This allows the comparison of variables according to similar induction conditions with the emphasis on the goal discrepancy to be activated in the unresolved goal condition but not in the resolved goal condition. However, the reflection of an achieved goal can initiate the processing of currently pursued, unachieved goals [66]. Consequently, information associated with unachieved goals may have higher availability, be prioritized accordingly, and thus automatically attract attention [67]. Therefore, to draw more precise conclusions about which conditions in the goal-cueing task should be used in further research, it is necessary to compare the two control conditions (resolved goal condition *vs*. neutral control condition) with each other as well as the effectivity of the shortened experimental condition compared to the original unresolved condition from Roberts and colleagues [3].

## Experiment 2

Experiment 2 aimed to address some of the limitations observed in Experiments 1a-c, namely, the lack of comparison between the original goal-related control condition of Roberts et al. [3] and the neutral control condition previously used in Experiment 1b and 1c. Further we aimed at comparing our experimental condition 1 (i.e., shortened audio instructions; 5 min), with the original unresolved experimental condition of Roberts et al. [3] (see for a detailed description *Measures and Material* section Experiment 1a). In detail, we compared the experimental condition 1 (i.e., shortened unresolved condition; EC1) and the neutral control condition (i.e., neutral control condition not related to a goal; NCC) from Experiment 1c with the original experimental condition from Roberts et al. [3, i.e., original unresolved condition; EC2] as well as the original resolved control condition from Roberts et al. [3, i.e., goal-related control condition; GRCC]. Using four 4 (EC1, EC2, GRCC, NCC) x 2 (before the goal-cueing task, after the goal-cueing task) mixed ANOVAs, we examined the effectiveness of the goal-cueing task with different state ruminations measures. For this purpose, in addition to the already applied measures (BSRI, ruminative self-focus index, and general rumination rating) we used a new measure to capture participant’s momentary repetitive negative thinking [64]. Although we criticized the use of the BSRI in Experiments 1a-c, we applied it again in Experiment 2 to be consistent with our previous experiments and to be able to directly compare results. Moreover, we assessed the same additional variables (MDMQ and perceived strain) to account for emotional processes in addition to cognitive processes, and again used the SART as an additional outcome measure.

### Methods

#### Participants

We performed an a priori power analysis (G*Power 3.1.) [68] for Experiment 2 to determine an appropriate sample size. To obtain a mean interaction effect (*f* = 0.25) with an alpha of α = .05 and a power of .80 using a mixed ANOVA with time (induction: pre vs. post) and condition (EG1 vs. EG2 vs. GRCC vs. NCC) as factors, a total sample size of *N* = 184 was recommended (n = 46 per condition).

Overall, 214 participants completed the experiment. However, we excluded two participants who reported not making a serious effort to follow the implementation instructions and further seven participants, who stated that they had already participated in a previous study on the same topic. Accordingly, the final sample consisted of 205 participants (female: n = 142; male: n = 61; divers: n = 2). The mean age was 24.28 (*SD* = 5.70). Most of the participants were students with different subjects (n = 172), followed by 21 employers. The remaining 12 participants were either pupils, trainees, civil servants, self-employed, job seekers or other. Table 6 presents an overview of sample characteristics separated by condition. Moreover, participants in the four conditions did not significantly differ in their level of trait rumination, *F*(1,203) = 0.07, *p* = .79, ƞp^2^ < .01.

**Table 6:** Mean (M), standard deviations (SD), 95% confidence intervals (95% CI) and Cronbach’s alpha (α) of sample characteristics separated by conditions for Experiment 2.

		Experimental condition 1		Experimental condition 2		Goal-related control condition		Neutral control condition	
	*α*	*M* (*SD*)	*95%CI*	*M* (*SD*)	*95%CI*	*M* (*SD*)	*95%CI*	*M* (*SD*)	*95%CI*
		n = 50 (f = 34, m = 15, d = 1)		n = 49 (f = 34, m = 15)		n = 53 (f = 39, m = 14)		n = 53 (f = 35, m = 17, d = 1)	
Age		23.72 (5.43)		24.31 (4.84)		24.28 (6.39)		24.77 (6.06)	
PTQ	.92	27.20 (10.96)	[24.08, 30.31]	29.47 (11.37)	[26.20, 32.74]	27.38 (9.36)	[24.80, 29.96]	28.47 (9.14)	[25.95, 30.99]
RSQ-B	.71	10.54 (3.03)	[9.68, 11.40]	11.67 (3.64)	[10.63, 12.72]	10.45 (2.98)	[9.63, 11.27]	10.11 (2.80)	[9.34, 10.89]
RSQ-R	.70	11.44 (3.08)	[10.56, 12.31]	11.26 (3.06)	[10.38, 12.14]	10.94 (3.01)	[10.11,11.77]	11.79 (3.22)	[10.90, 12.68]
GSE	.85	28.98 (4.78)	[27.62, 30.34]	28.43 (4.82)	[27.04, 29.81]	28.40 (4.51)	[27.15, 29.64]	29.90 (4.16)	[28.76, 31.05]

*Note*. n = number of participants, f = female, m = male, PTQ = Perseverative Thinking Questionnaire, RSQ-B = Brooding subscale of Response Styles Questionnaire, RSQ-R = Reflection subscale of the Response Styles Questionnaire, GSE = General Self-Efficacy Scale.

#### Evaluation of the quality of the online experiment

Participants rated the quality of their performance, indicating that their concentration was 3.48 (*SD* = 1.01), that they made an average effort of 4.46 (*SD* = 0.68) to follow the instructions seriously, and that they succeeded with 4.02 (*SD* = 0.92). In terms of concentration, there were no differences between participants in the four conditions, *F*(3,201) = 1.61, *p* = .19, ƞp^2^ = .02. In contrast, GRCC (*M* = 4.25, *SD* = 0.72) differed significantly from NCC (*M* = 4.68, *SD* = 0.55; *p*_bonf_ < .01) in how seriously they tried to perform the experiment, *F*(3,201) = 4.14, *p* = .01, ƞp^2^ = .06. In addition, there was also a significant difference between NCC (*M* = 4.32, *SD* = 0.85) and GRCC (*M* = 3.83, *SD* = 0.89; *p*_bonf_ = .03) in terms of how well they succeeded in implementing the instructions, *F*(3,201) = 3.45, *p* = .02, ƞp^2^ = .05.

#### Procedure

Participants were invited to participate in the experiment via institutes’ student mailing list, via the respective study offices of the study programs as well as notices on the bulletin board of the Johannes Gutenberg-University Mainz, and personal contacts. Participation in this experiment was voluntary and participants received 12 Euro as compensation (psychology students could alternatively receive course credit for their participation). Otherwise, the procedure is the same as in the Experiments 1a-c.

#### Measures & material

Here we report only the measures that we added compared to Experiments 1a-c.

*State Rumination*. In addition to the already used BSRI, ruminative self-focus index, and the general rumination rating, we applied a measure to capture momentary repetitive negative thinking (MRNT, i.e., process-related scale) [64]. The scale comprises four items (e.g., “Thoughts come to my mind without me wanting them to”.), each of which focused on a core characteristic of rumination–repetitiveness, intrusiveness, uncontrollability, and impairment. Participants answer each statement on a 7-point scale ranging from ‘1’ (*not at all*) to 7 (*very*). Just like the BSRI, we collected the MRNT at four points in time.

*Goal-cueing task*. EC1 and NCC remained the same as in Experiment 1c. EC2 and GRCC referred to the original conditions of Roberts and colleagues [3, for a detailed description of EC2 see Measures & Materials section of Experiment 1a]. Participants in the GRCC were instructed to identify a problem from the past that has since been resolved, has not come to mind in the past, and no longer makes them feel bad, sad, down, or stressed. Like EC2, participants were asked to briefly outline the identified problem in writing, answer some questions about it, and follow a 10-minute audio instruction (for example: "Think about why solving this problem will make progress toward your personal goals; for detailed information see Roberts et al. [3]).

#### Data analyses

We performed the same analysis as in Experiments 1c. The only difference is that we used four conditions in the group comparisons. In case of a significant two-way interaction, we applied a simple main effect analysis (one-way model) of the condition variable at each level of the time variable and reported adjusted *p*-values according to Bonferroni. If the simple main effect was significant, we ran multiple pairwise comparisons to determine which conditions were different. In addition, differences in terms of goal characteristics and post evaluation were calculated using an ANOVA rather than independent *t*-tests due to the three conditions being compared. Moreover, we also computed the same exploratory analyses in terms of the influence of gender as well as the change in the state measures during the SART. The results of these analyses are summarized in S4 to S6 Tables.

### Results

#### Characterization of problems in the individual goal achievement process

Results indicated that there were significant differences between the conditions in five of six goal-related questions. There was no significant effect of condition on participants’ evaluation of how long the problem exists. The upper part of Table 7 summarizes the descriptive statistics and the results of the *F*-tests for participants’ goal-evaluations. Post-hoc tests showed that both ECs differed significantly from GRCC in terms of trouble at the time of the experiment and time spent on the problem (both *p*_bonf_ < .001).

**Table 7:** Mean (M), standard deviations (SD), test statistics, and effect sizes of participants’ goal characteristics and goal evaluations after the experiment.

	Experimental condition 1		Experimental condition 2		Goal-related control condition		Results *F*-statistic	Effect size (ηp^2^)
*Goal characteristics*	*M*	*SD*	*M*	*SD*	*M*	*SD*		
(1) Trouble at the time of the experiment	7.92	1.81	7.61	1.90	1.74	2.16	*F*(2,149) = 162.90, *p* < .001	.69
(2) Trouble in its worst…	9.02	1.15	8.67	1.43	8.17	1.73	*F*(2,149) = 4.40, *p* = .01	.05
(3) Goal Importance	8.58	1.49	8.29	1.53	7.58	1.78	*F*(2,149) = 5.23, *p* < .01	.06
(4) Example for general problems	6.34	2.41	5.92	2.33	5.11	2.59	*F*(2,149) = 3.34, *p* = .04	.04
(5) Duration of the problem	6.28	2.25	5.71	2.92	5.13	2.82	*F*(2,149) = 2.37, *p* = .10	.03
(6) Time spent with the problem	7.10	2.56	6.59	2.07	3.79	3.18	*F*(2,149) = 23.26, *p* < .001	.24
***Goal evaluation***								
(1) Difficulties stop thinking about the problem	2.66	1.27	2.55	1.14	2.26	1.23	*F*(2,149) = 1.47, *p* = .23	.02
(2) Problem seemed worse	2.54	1.28	2.71	1.08	2.51	1.27	*F*(2,149) < 1	-
(3) Participant felt worse	2.86	1.11	3.06	1.39	2.64	1.33	*F*(2,149) = 1.37 *p* = .26	.02
(4) Focus on negative aspects	3.00	1.28	3.31	1.19	2.70	1.29	*F*(2,149) = 2.98, *p* = .05	.04
(5) Focus on bad feelings	2.66	1.22	2.94	1.23	2.66	1.25	*F*(2,149) < 1	-

*Note*. Experimental condition 1 = shortened unresolved condition (5min goal focus period), Experimental condition 2 = original unresolved condition.

In addition, only the EC1 significantly differed from the GRCC in terms of goal importance (*p*_bonf_ < .01), how much the goal related to general concerns (*p*_bonf_ = .04), and how much the problem had bothered them at its worst (*p*_bonf_ = .01). Thus, the goals identified in the two ECs did not differ in subjective evaluations of their nature or severity, but participants in the ECs (especially in the EC1) reported that the problem was one that was bothering them more than participants in the GRCC.

#### Effects of goal-cueing task on state rumination, mood, and perceived strain

Four two-factorial mixed effects ANOVAs with the between group factor condition (4 levels) and the repeated measure factor time (2 levels: pre vs. post goal-cueing task) were computed to examine the effects of the goal-cueing task on state rumination, mood, and perceived strain. Table 8 presents the mean values and standard deviations of the relevant experimental variables. With regard to state rumination, results indicated that there were significant interaction effects and significant condition effects for all used measures. For time, however, only two of four state rumination measures showed a significant effect.

**Table 8:** Mean values and standard deviations of relevant experimental variables separated by condition and time for Experiment 2.

	Experimental condition 1		Experimental condition 2		Goal-related control condition		Neutral control condition	
	Pre	Post	Pre	Post	Pre	Post	Pre	Post
*State Rumination*	*M* (*SD*)	*M* (*SD*)	*M* (*SD*)	*M* (*SD*)	*M* (*SD*)	*M* (*SD*)	*M* (*SD*)	*M* (*SD*)
MRNT	3.84 (1.52)	4.18 (1.61)	3.84 (1.49)	4.22 (1.44)	3.64 (1.52)	3.30 (1.23)	3.57 (1.45)	2.81 (1.58)
BSRI	3.44 (2.15)	4.10 (2.50)	3.85 (2.47)	4.95 (2.28)	3.58 (1.80)	4.01(1.93)	3.36 (2.06)	2.77 (2.12)
Ruminative self-focus	4.33 (1.46)	5.21 (1.29)	4.31 (0.86)	5.41 (1.16)	4.31 (1.23)	4.86 (1.06)	4.47 (1.03)	3.07 (1.10)
General rumination rating	4.42 (1.69)	5.20 (1.24)	4.26 (1.47)	4.98 (1.39)	4.06 (1.69)	4.07 (1.35)	4.23 (1.68)	2.98 (1.73)
***Mood***								
Energetic Arousal	2.80 (1.30)	2.78 (1.33)	3.71 (1.25)	3.25 (1.24)	3.47 (1.01)	3.37 (1.07)	3.29 (1.17)	3.42 (1.24)
Valence	3.53 (1.10)	2.88 (1.14)	3.89 (1.12)	3.12 (1.29)	3.80 (1.12)	3.57 (1.16)	3.62 (1.07)	3.94 (1.24)
Calmness	3.54 (1.29)	3.27 (1.19)	3.64 (1.22)	3.16 (1.31)	3.74 (1.15)	3.67 (1.12)	3.76 (1.20)	4.13 (1.07)
***Perceived strain***	4.16 (1.61)	4.96 (1.46)	4.08 (1.51)	4.90 (1.49)	3.83 (1.65)	3.28 (1.25)	4.09 (1.57)	2.77 (1.68)

*Note*. Experimental condition 1 = shortened unresolved condition (5min goal focus period), Experimental condition 2 = original unresolved condition, pre = measurement before the goal-cueing task, post = measurement after the goal-cueing task, BSRI = Brief State Rumination Inventory, MRNT = Momentary repetitive negative thinking index.

*BSRI*. There was a significant main effect of condition, *F*(3,201) = 3.86, *p* = .01, ηp^2^ = .05, and a significant main effect of time, *F*(1,201) = 11.22, *p* = .001, ηp^2^ = .05 which were qualified by a significant interaction between time and condition, *F*(3,201) = 8.94, *p* < .001, ηp^2^ = .12. Subsequent pairwise comparisons between condition levels showed that the simple main effect of condition was not significant for the time prior the goal-cueing task (*p*_bonf_ = .67) but for the time after to the goal-cueing task (*p*_bonf_ < .001). Pairwise comparison between post goal-cueing task and each condition level showed that the mean BSRI score was significantly different in EC1 vs. NCC (*p*_bonf_ = .01), in EC2 vs. NCC (*p*_bonf_ < .001) and in CC vs. NCC (*p*_bonf_ = .02).

*Momentary repetitive negative thinking*. With regard to the results of MRNT, a significant condition effect was obtained, *F*(3,201) = 4.75, *p* < .01, ηp^2^ = .07, as well as a significant time x condition interaction, *F*(3,201) = 11.67, < .001, ηp^2^ = .15. The effect for time was not significant, *F*(1,201) = 1.35, *p* = .25, ηp^2^ = .01. Considering the Bonferroni-adjusted *p*-value of the simple main effect analysis, the effect for condition at the time before the goal-cueing task was not significant (*p*_bonf_ = .44) compared to the time after the goal-cueing task (*p*_bonf_ < .001). Pairwise comparison showed that the MRNT was significantly different in EC1 vs. NCC (*p*_bonf_ < .001), in EC2 vs. NCC (*p*_bonf_ < .001), as well as in EC1 vs. GRCC (*p*_bonf_ = .01) and in EC2 vs. GRCC (*p*_bonf_ = .01).

*Ruminative self-focus*. There was a significant main effect of condition, *F*(3,201) = 16.54, *p* < .001, ηp^2^ = .20, and a significant main effect of time, *F*(1,201) = 7.46, *p* < .01, ηp^2^ = .03, which were qualified by a significant interaction between time and condition, *F*(3,201) = 29.96, *p* < .001, ηp^2^ = .31. Subsequent pairwise comparisons between condition levels showed that the simple main effect of condition was not significant for the time prior the goal-cueing task (*p*_bonf_ = .87) but for the time after to the goal-cueing task (*p*_bonf_ < .001). Pairwise comparison between post goal-cueing task and each condition level showed that ruminative focus differed in EC1 vs. NCC (*p*_bonf_ < .001), in EC2 vs. NCC (*p*_bonf_ < .001) and in GRCC vs. NCC (*p*_bonf_ < .001).

*General rumination rating*. Regarding the general state rumination rating, a significant effect of condition, *F*(3,201) = 9.88, *p* < .001, ηp^2^ = .13, as well as of the interaction between time and condition, *F*(3,201) = 13.82, *p* < .001, ηp^2^ = .17 was obtained. The effect for time was not significant, *F*(1,201) < 1. Results of the subsequent simple main effect analysis showed that the effect for condition at the time before the goal-cueing task was not significant (*p*_bonf_ = .73) but was significant at the time after the goal-cueing task (*p*_bonf_ < .001). Pairwise comparison showed that the general rumination rating was different in participants from EC1 vs. NCC (*p*_bonf_ < .001), in EC2 vs. NCC (*p*_bonf_ < .001) and in GRCC vs. NCC (*p*_bonf_ < .001). There were also significant differences between EC1 vs. GRCC (*p*_bonf_ < .001) and EC2 vs. GRCC (*p*_bonf_ = .01).

*Mood*. We conducted three more 4 × 2 ANOVAs to also examine the effects of the goal-cueing task on mood assessed by the MDMQ. Regarding the energetic arousal, there was a significant effect for condition, *F*(3,201) = 4.02, *p* < .01, ηp^2^ = .05, and for the time x condition interaction, *F*(3,201) = 4.61, *p* < .01, ηp^2^ = .06, as well as a tendency to significance for time, *F*(1,201) = 3.77, *p* = .05, ηp^2^ = .02. Subsequent pairwise comparisons between condition levels showed that the simple main effect of condition was significant for the time prior the goal-cueing task (*p*_bonf_ < .01) but not for the time after to the goal-cueing task (*p*_bonf_ = .07). Pairwise comparison between pre goal-cueing task and each condition level showed that energetic arousal differed in EC1 vs. EC2 (*p*_bonf_ = .001) as well as in EC1 and GRCC (*p*_bonf_ = .03). With regard to valence, we found a significant effect of condition, *F*(3,201) = 3.09, *p* = .03, ηp^2^ = .04, and a significant main effect of time, *F*(1,201) = 20.69, *p* < .001, ηp^2^ = .09 which were qualified by a significant interaction between time and condition, *F*(3,201) = 11.36, *p* < .001, ηp^2^ = .14. Subsequent main effect analysis of the condition variable revealed no significant differences before the goal-cueing task (*p*_bonf_ = .70), but significant differences after the goal-cueing task (*p*_bonf_ < .001). Pairwise comparison showed that valence was different in participants from EC1 vs. NCC (*p*_bonf_ < .001), in EC2 vs. NCC (*p*_bonf_ < .01), and EC1 vs. GRCC (*p*_bonf_ = .03). Finally, results of the 4 x 2 ANOVA for calmness indicated a significant effect for condition, *F*(3,201) = 3.04, *p* = .03, ηp^2^ = .04, and for the time x condition interaction, *F*(3,201) = 6.77, *p* < .001, ηp^2^ = .09, but not for time, *F*(1,201) = 2.70, *p* = .11, ηp^2^ = .01. Subsequent pairwise comparisons between condition levels showed that the simple main effect of condition was not significant for the time prior the goal-cueing task (*p*_bonf_ = .99) but for the time after to the goal-cueing task (*p*_bonf_ < .001). Pairwise comparison showed that calmness values were different in participants from EC1 vs. NCC (*p*_bonf_ = .001), and in EC2 vs. NCC (*p*_bonf_ < .001).

*Perceived strain*. Last, a 4 × 2 mixed ANOVA was applied to investigate the effect of the goal-cueing task on the perceived strain of the participants. There was a significant effect of condition, *F*(3,201) = 11.68, *p* < .001, ηp^2^ = .15, and a significant effect of the interaction between time and condition, *F*(3,201) = 18.14, *p* < .001, ηp^2^ = .21. The effect for time was not significant, *F*(1,201) < 1. Subsequent pairwise comparisons between condition levels showed that the simple main effect of condition was not significant for the time prior the goal-cueing task (*p*_bonf_ = .99) but for the time after to the goal-cueing task (*p*_bonf_ < .001). Pairwise comparison between post goal-cueing task and each condition level showed that perceived strain differed in EC1 vs. NCC (*p*_bonf_ < .001), in EC2 vs. NCC (*p*_bonf_ <. 001), as well as in EC1 vs. GRCC (*p*_bonf_ < .001) and in EC2 vs. GRCC (*p*_bonf_ < .001).

#### Errors of commission and mean RT during the SART

Table 9 illustrates the percentage error rates and mean RTs for each condition during each block of the SART and the respective results of the *F*-statistic. Regarding participants errors of commission, results revealed a significant effect for condition, *F*(3,187) = 3.30, *p* = .02, ηp^2^ = .05, and for time, *F*(1,187) = 7.36, *p* < .01, ηp^2^ = .04, but not for the interaction between condition x time, *F*(3,187) = 2.04, *p* = .11, ηp^2^ = .03. With regard to the mean RT for correct go-trials, there was significant effect for time, *F*(1,187) = 6.26, *p* = .01, ηp^2^ = .03, but no significant effects for condition as well as for the condition x time interaction, both *F*’s(3,187) < 1, indicating slower mean RTs as time went on.

**Table 9:** Mean values (M) and standard deviations (SD) of errors of commission (in percent) and mean RTs for correct go-trials during the Sustained Attention to Response task (SART).

	Block 1	Block 2	Results *F*-statistic	
	*M (SD)*	*M (SD)*	Errors of commission	Mean RT
***Experimental condition 1***			*F*_C_(3,187) = 3.30, *p* = .02, ηp^2^ = .05 *F*_T_(1,187) = 7.36, *p* < .01, ηp^2^ = .04 *F*_I_(3,187) = 2.04, *p* = .11, ηp^2^ = .03	*F*_C_(3,187) < 1 *F*_T_(1,187) = 6.26, *p* = .01, ηp^2^ = .03 *F*_I_(3,187) < 1
Errors of commission	51.48 (17.08)	58.96 (22.27)		
Mean RT	377.35 (71.88)	390.32 (94.19)		
***Experimental condition 2***				
Errors of commission	46.42 (21.29)	47.28 (22.32)		
Mean RT	395.02 (74.63)	409.23 (108.25)		
***Goal-related control condition***				
Errors of commission	42.33 (20.79)	44.04 (22.42)		
Mean RT	405.03 (86.51)	411.65 (89.73)		
***Neutral control condition***				
Errors of commission	44.64 (19.34)	46.16 (22.51)		
Mean RT	386.56 (60.07)	396.13 (80.29)		

*Note*. Experimental condition 1 = shortened unresolved condition (5min goal focus period), Experimental condition 2 = original unresolved condition, Mean RT = Mean reaction time; *F*_C_ = *F*-statistic for condition effect, *F*_T_ = *F*-statistic for time effect, *F*_I_ = *F*-statistic for interaction effect. We excluded data from fifteen participants due to no serious reactions during the SART (valid go-trials = 0).

#### Evaluation of the identified problem/concern after the experimental setting

No differences were found between the EC1, EC1 and the GRCC regarding the evaluation of the identified problems after the experiment (see Table 7). We also asked participants in both control conditions if they were thinking about a current problem that was bothering them during the experiment. This question was answered in the affirmative by 40 participants from GRCC (75.5%) and 31 participants from NCC (58.5%).

### Discussion Experiment 2

Experiment 2 had two aims. First, we aimed at testing which control condition (goal-related *vs*. neutral) is best suited to maximize the effects of the goal-cueing task in terms of state rumination in future research. Second, we wanted to examine whether the shortened unresolved condition is as effective in inducing state rumination as the original unresolved condition by Roberts et al. [3]. For this purpose, we applied different rumination measures, namely the BSRI, the ruminative self-focus index, a general rumination rating, and a momentary repetitive negative thinking measure and performed four two-factorial mixed effects ANOVAs. Moreover, we assessed further variables (MDMQ and perceived strain), using the same approach, to account for emotional processes in addition to cognitive processes and again used the SART as an additional outcome measure.

Results of the mixed ANOVA indicated a successful induction of rumination, which is expressed by significant interaction effects (time x condition) on all four state rumination measures. Subsequent pairwise comparisons between condition levels showed that the simple main effect of condition was not significant for the time prior the goal-cueing task but was significant for the time after the goal-cueing task. This was consistent for all applied state rumination measures. In detail, participants in the two experimental conditions reported significantly higher scores on all four state ruminations measures after the goal-cueing task (except for the BSRI for the EC1, which showed only a trend effect with *p*_bonf_ = .06) compared to participants from the neutral control condition. However, this did not apply to the goal-related control condition (i.e., original resolved condition by Roberts et al. [3]). Here, only the examination of the general rumination rating showed a significant difference to EC1 after the goal-cueing task. In addition, the GRCC showed higher state ruminations scores on two of four measures (ruminative self-focus, general ruminations rating) compared to the NCC. This could also be partly because 75% of the participants in the GRCC also thought about a current problem while conducting the experiment. According to Shah [67], reflecting on a solved problem during the goal-focusing period may have resulted in initiating the processing of currently pursued, unachieved goals. Consequently, it cannot be assumed that the goal discrepancy is activated only in the unresolved goal condition and not in the resolved goal condition. Whether these results ultimately also had an influence on the quality of the execution of the experiment, however, remains open. Here, minor differences between the two control conditions (*M*_Δ_ < 0.50) emerged in the sense that the goal-related control condition reported lower values for seriousness and successful execution. To summarize, in terms of state rumination, the results of Experiment 2 showed that the two ECs can be used equally well to induce state rumination in participants, which means that depending on the experimental design and the time available, either the original experimental condition or the shortened experimental condition can be applied. To maximize the effects in terms of state rumination, the NCC seems to be more suitable due to the better differentiation in the state measures.

In terms of mood, valence in EC2 was found to decrease during the goal-cueing task, and participants in both ECs reported significantly lower valence scores thereafter compared to the NCC. It seems unsurprising that participants who are asked to address an unresolved issue that has made them sad, depressed, or down in the recent past feel more uncomfortable than someone who is asked to be as objective as possible about their past daily routine. In addition, the NCC participants showed higher calmness values after the goal-cueing task compared to both ECs and the effect of energetic arousal refers to the difference in pre induction values between EC1 and GRCC and the two ECs. With this one exception, no further significant differences were found between the GRCC and the two ECs. Accordingly, it can be assumed that the mood in both ECs is similar, in some cases slightly more "negative" compared to the NCC, but not compared to the GRCC. The latter is consistent with Roberts et al. [3], who showed that participants in the unresolved and resolved condition did not differ with respect to sadness, tension, and self-focus. However, in the subsequent study [32], the two conditions were found to have different effects on participants’ negative affect (measured with the Positive Affect and Negative Affect Schedule [69], in the sense that negative affect increased during the goal-cueing task in the unresolved condition and differed significantly from the resolved condition thereafter. So far, however, it seems difficult to draw conclusions about the change in mood during the goal-cueing task, as either mood or affect were assessed with different measures in the studies.

In terms of performance in the SART, participants’ reaction times worsened regardless of condition and more errors were made as time went on. A condition effect indicates that the EC1 in principle has a higher error rate in the SART compared to the other conditions. However, due to the lack of interaction effects, no significant differences can be found with respect to the goal-cueing task. Why no other significant main effects were obtained in the SART is discussed in the general discussion.

Finally, we would like to address a potential limitation of this study: To examine the effectiveness of the goal-cueing task and its directly observable effect on state rumination, we used different state rumination measures to capture rumination with focus on different aspects (e.g., core characteristics of rumination, self-focused attention, attention to one’s distress and its possible causes and effects, general state of rumination), and to figure out which measure would be most appropriate for future studies. However, it cannot be ruled out that these in some way influenced each other. Therefore, the number of measurement variables for rumination should be reduced in future studies to avoid potential interference.

### General discussion

The aim of the four experiments of the present study was to evaluate the effectivity of the goal-cueing task procedure [3] to induce state rumination on an individual level in different experimental settings. For this, we used different measures of state rumination covering different facets of rumination, namely the BSRI [23], an index of ruminative self-focus [15], a general rumination rating [37], and an EMA measure of momentary repetitive negative thinking [64]. Specifically, we were interested in whether the use of the goal-cueing task had a direct observable effect on state rumination as assessed by these different state rumination measures.

Since we have already discussed the results of each of the individual experiments above, we would only like to give a brief overview of the main results and discuss overarching aspects in this section. Experiment 1a showed that shortening the instructions of the unresolved condition (i.e., omitting step 3 –goal focus period) did not in principle affect the results. However, the significance is limited because a control condition was missing to relate the effects found. In Experiment 1b, we compared the EC from the previous experiment with two further conditions: another shortened experimental condition (included only step 1 of the goal-cueing task) and a neutral control condition that was not related to a personal goal. Here, for the first time, we found a significant interaction between time and condition with respect to the general rumination rating. However, Experiment 1b was severely limited by the small sample size and the general homogeneous sample of psychology students. Experiment 1c picked up the shortened experimental condition from Experiment 1a and compared it with another shortened experimental condition (reduced goal-focus period to 5 min) and the neutral control condition. By shortening the goal focus period, previously existing concerns about the processing style of rumination during the goal-cueing task could be eliminated. Significant interaction effects on both state rumination measures underlined the assumption of an effective form of rumination induction. Finally, Experiment 2 filled the gap, which became evident by the limitations and open questions of the previous experiments: 1) the comparison of the experimental condition with the shortened goal focus period and the neutral control condition from Experiment 1c with the original experimental condition from Roberts et al. [3, i.e., original unresolved condition] as well as the original resolved control condition from Roberts et al. [3, i.e., goal-related control condition]. Results from Experiment 2 confirmed a successful induction of rumination through the shortened experimental condition, which is expressed by significant interaction effects on all four state rumination measures.

Regarding the performance in the SART, apart from a few time effects (and an additional condition effect in Experiment 2); there were no relevant rumination-related effects. Null results between conditions regarding the SART may be attributed to three different reasons: First, consistent with the findings of Roberts et al. [32], the absence of condition effects could indicate that participants in the conditions did not differ in their attentional control capabilities. This is in line with a study by Schmitz-Hübsch and Schindler [70] who could not replicate the postulated relationship between performance on the SART in two different versions and self-reported everyday errors. Further, a study by Edwards [34] did not find differences in SART performance between a goal-avoidance and a goal-approach condition following the goal-cueing task. Second, participants in the unresolved condition, due to the perceived goal discrepancy, might have used more resources, such as attention, effort, or energy to solve the current discrepancy accordingly [71–73], and thus obtained similar results in the SART as the participants in the control conditions. Roberts et al. [3] even indicated that participants who focused on unresolved goals were slower and more accurate on the SART compared to participants in the resolved condition and speculated that this might be a compensatory strategy of slowing responses to reduce the risk of errors. Accordingly, the SART could also be seen as a welcome distraction to switch from a self-referential focus to an external focus. Third, Huffziger and colleagues [16] suggested that consequences of induced rumination might be stronger when inductions occur in natural contexts because it produces more personal-relevant reasons to ruminate about, which may consequently influence individuals’ actions and behaviors. Following the three reasons mentioned above, the use of the SART should be reconsidered in future studies depending on the research question.

Furthermore, in our own as well as previous studies [3,32,34], participants defined a personally relevant goal but were then confronted with a task that was unrelated to the defined goal and thus did not seem goal enhancing or particularly personally relevant. Accordingly, the question remains open how the goal manipulation affects the performance of personally relevant tasks such as they may occur in everyday life, for example perceived discrepancy or problems at work [74], an exam at university [8], or competitions in sports [75]. Future studies could therefore combine the goal-cueing task with a more relevant task in the laboratory or directly transfer the goal-cueing task into everyday life by means of an ambulatory assessment approach (see for example Huffziger et al. [16]). For this, the different modifications of our experimental conditions could provide first interesting starting points.

Finally, successful, and valid induction of rumination could be particularly useful in explaining causal relationships between rumination and individual goal achievement (e.g., what types of goals are more likely to trigger rumination that potentially affect daily performance and impact well-being, are they avoidance or approach goals, well versus poorly defined goals, or externally motivated goals versus internally motivated goals). Moreover, as a tool, this induction form can support further research in uncovering the mechanisms underlying rumination that influence specific mental processes (e.g., testing further hypotheses from the H-EX-A-GO-N model [1]). Building on the findings, future research could develop specific cognitive interventions (e.g., adapting cognitive-behavioral therapy for rumination for a nonclinical context [see: 24]) and effective problem-solving strategies through which rumination could be resolved and prevented in the future.

### Limitations

A general limitation was that Experiments 1a-c took place during pandemic lockdown and therefore were performed as online experiments (Experiment 2 was then also conducted as an online experiment to be consistent with the previous experiments), making it much more difficult to control conditions and potential confounding variables compared to conducting the experiments in the laboratory [39]. However, we took some steps to increase the quality of the implementation of the online experiments (compare *Advantage and disadvantage of applying an online experiment in our study* section Experiment 1a). In addition, we asked the participants for their honest evaluation of their performance quality and the results suggested that the implementation and the environment was appropriate for participation in the experiments. In addition, existing comparative studies between laboratory and online experiments showed that the results agree surprisingly well [76,77].

### Conclusion

In conclusion, the current experiments add to the research field of rumination and goal achievement by confirming experimental evidence for the correlation of rumination and individual goal achievement in the face of unresolved goals. Ultimately, these findings contribute to our understanding of potential triggers for rumination in non-clinical samples. Thus, the goal-cueing task allows researchers to test causal links between rumination and factors that are thought to lead to ruminative thinking. In addition, the results of the four experiments provide hints as to how the paradigm can be modified to be applied in other experimental settings in and outside the laboratory. In the future, the effects potentially achieved with this paradigm could also be significant in real life. For example, it would be valuable to examine the relationship between the dynamics of momentary rumination and day-to-day performance, as well as performance in specific contexts (e.g., work, sport, academics etc.).

## Supporting information

S1 Table Results of the multivariate analysis with group, gender and measure time as well as their respective interaction as factors and different rumination measures as dependent variables for Experiments 1a-c. (PDF)

S2 Table Test statistics for variables during the SART and corresponding effect sizes (ηp^2^) of the respective mixed ANOVAs separated by effects for Experiments 1a-c. (PDF)

S3 Table Mean values and standard deviations of relevant experimental variables assessed during the SART and separated by condition and time for Experiments 1a-c. (PDF)

S4 Table Results of the multivariate analysis with group, gender and measure time as well as their respective interaction as factors and different rumination measures as dependent variables for Experiment 2. (PDF)

S5 Table Test statistics for variables during the SART and corresponding effect sizes (ηp^2^) of the respective mixed ANOVAs separated by effects for Experiment 2. (PDF)

S6 Table Mean values and standard deviations of relevant experimental variables assessed during the SART and separated by condition and time for Experiments 2. (PDF)
