# Supplementary material for: Experimental induction of state rumination: A study evaluating the efficacy of goal-cueing task in different experimental settings
Source: PLoS One. 2023 Nov 22;18(11):e0288450. doi: 10.1371/journal.pone.0288450 (PMC10664951; doi:10.1371/journal.pone.0288450)
Supplement: S1 Table — (PDF) [file pone.0288450.s001.pdf]

Table S1

Results of the multivariate analysis with group, gender and measure time as well as their respective interaction as factors and different rumination measures as dependent variables for Experiments 1a-c.

	df	*F*-value	*p*-value	ηp^2
Exp. 1a				
**BSRI**				
group	1,160	<1	.80	<.01
gender	1,160	<1	.60	<.01
measure_time	1,160	27.91	<.001	.15
group:gender	1,160	<1	.37	<.01
group:measure_time	1,160	1.63	.20	.01
gender:measure_time	1,160	1.12	.29	<.01
group:gender:measure_time	1,160	<1	.77	<.01
**General rumination rating**				
group	1,160	1.90	.17	.01
gender	1,160	< 1	.94	< .01
measure_time	1,160	65.31	< .001	.29
group:gender	1,160	1.78	.18	.01
group:measure_time	1,160	6.30	.01	.04
gender:measure_time	1,160	3.73	.05	.02
group:gender:measure_time	1,160	< 1	.43	< .01
Exp. 1b				
**BSRI**				
group	2,33	1.38	.26	.07
gender	1,33	<1	.99	< .01
measure_time	1,33	7.39	.01	.18
group:gender	2,33	2.58	.09	.13
group:measure_time	2,33	1.39	.26	.08
gender:measure_time	1,33	1.83	.18	.05
group:gender:measure_time	2,33	< 1	.97	< .01
**General rumination rating**				
group	2,33	9.12	< .001	.36
gender	1,33	1.68	.20	.05
measure_time	1,33	8.75	< .01	.21
group:gender	2,33	3.63	.04	.18
group:measure_time	2,33	5.22	.01	.24
gender:measure_time	1,33	< 1	.47	.01
group:gender:measure_time	2,33	3.13	.06	.16

Table S1 continued

	df	*F*-value	*p*-value	ηp²
Exp. 1c				
**BSRI**				
group	2,53	< 1	.44	.03
gender	1,53	< 1	.60	< .01
measure_time	1,53	2.44	.12	.04
group:gender	2,53	< 1	.57	.02
group:measure_time	2,53	11.27	< .001	.30
gender:measure_time	1,53	5.33	.02	.09
group:gender:measure_time	2,53	< 1	.44	.03
**Ruminative Self-focus**				
group	2,53	2.34	.11	.08
gender	1,53	< 1	.49	< .01
measure_time	1,53	13.76	< .001	.21
group:gender	2,53	< 1	.57	.02
group:measure_time	2,53	11.02	< .001	.29
gender:measure_time	1,53	1.55	.22	.03
group:gender:measure_time	2,53	< 1	.45	.03

Note. BSRI = Brief State Rumination Inventory (Marchetti et al., 2018); *df* = degrees of freedom, ηp² = partial eta squared (effect size)
