# Supplementary material for: Experimental induction of state rumination: A study evaluating the efficacy of goal-cueing task in different experimental settings
Source: PLoS One. 2023 Nov 22;18(11):e0288450. doi: 10.1371/journal.pone.0288450 (PMC10664951; doi:10.1371/journal.pone.0288450)
Supplement: S2 Table — (PDF) [file pone.0288450.s002.pdf]

Table S2

Test statistics for variables during the SART and corresponding effect sizes (ηp^2) of the respective mixed ANOVAs separated by effects for Experiments 1a-c.

	Condition effect		Time effect		Interaction effect	
	Results *F*-statistic	ηp^2	Results *F*-statistic	ηp^2	Results *F*-statistic	ηp^2
Exp. 1a						
State Rumination						
General rumination rating	$F(1,162) < 1$	-	$F(3,486) = 1.86$, $p[GG] = .14$	.01	$F(3,486) = 1.92$, $p[GG] = .13$	.01
Mood						
Energetic Arousal	$F(1,162) < 1$	-	$F(3,486) = 38.16$, $p[GG] < .001$	.19	$F(3,486) < 1$	-
Valence	$F(1,162) = 1.01$, $p = .32$	.01	$F(3,486) = 9.87$, $p[GG] < .001$	.06	$F(3,486) = 1.08$, $p[GG] = .35$	.01
Calmness	$F(1,162) < 1$	-	$F(3,486) = 11.99$, $p[GG] < .001$	<.01	$F(3,486) < 1$	-
Perceived strain	$F(1,162) = 2.60$, $p = .11$	.02	$F(3,486) = 2.56$, $p[GG] = .06$	.01	$F(3,486) < 1$	-
Exp. 1b						
State Rumination						
General rumination rating	$F(2,36) < 1$		$F(3,108) = 3,18$, $p = .03$	.08	$F(6,108) < 1$	
Mood						
Energetic Arousal	$F(2,36) < 1$	-	$F(3,108) = 9.90$, $p[GG] < .001$	.21	$F(6,108) = 1.16$, $p[GG] = .34$	.06
Valence	$F(2,36) < 1$	-	$F(3,108) = 2.75$, $p[GG] = .06$	.07	$F(6,108) = 1.81$, $p[GG] = .12$	.09
Calmness	$F(2,36) < 1$	-	$F(3,108) = 3.14$, $p[GG] = .04$	.08	$F(6,108) < 1$	-
Perceived strain	$F(2,36) < 1$	-	$F(3,108) = 4.24$, $p[GG] = .02$	.10	$F(6,108) < 1$	-
Exp. 1c						
State Rumination						
Ruminative self-focus	$F(2,56) < 1$	-	$F(3,168) = 1.53$, $p[GG] = .21$	.03	$F(6,168) < 1$	-
Mood						
Energetic Arousal	$F(2,56) < 1$		$F(3,168) = 16.33$, $p[GG] < .001$	.22	$F(6,168) = 3.69$, $p[GG] <.01$	.12
Valence	$F(2,56) = 1.49$, $p = .23$	.05	$F(3,168) = 5.42$, $p < .01$	.09	$F(6,168) = 1.07$, $p =.38$	.04
Calmness	$F(2,56) = 1.98$, $p = .15$	.07	$F(3,168) < 1$	-	$F(6,168) < 1$	-
Perceived strain	$F(2,56) = 1.14$, $p = .32$	.04	$F(3,168) = 2.46$, $p = .06$	.04	$F(6,168) < 1$	-

Note. Exp. = experiments, *p[GG]* = *p*-value was corrected with Greenhouse-Geisser correction.
