# Supplementary material for: Experimental induction of state rumination: A study evaluating the efficacy of goal-cueing task in different experimental settings
Source: PLoS One. 2023 Nov 22;18(11):e0288450. doi: 10.1371/journal.pone.0288450 (PMC10664951; doi:10.1371/journal.pone.0288450)
Supplement: S3 Table — (PDF) [file pone.0288450.s003.pdf]

In order to interpret the time effects of the different mixed ANOVAs, the means and standard deviations of the state measures per condition and per measurement time for each experiment are presented below.

Table S3

Mean values and standard deviations of relevant experimental variables assessed during the SART and separated by condition and measurement time for Experiments 1a-c.

	**During SART: state measures**			
	t_1	t_2	t_3	t_4
**Exp.1a**				
General rumination rating				
EC1	2.03 (2.34)	2.74 (2.77)	1.99 (2.29)	2.37 (2.50)
EC2	2.08 (2.48)	2.04 (2.17)	2.00 (2.21)	1.99 (2.32)
Energetic Arousal				
EC1	3.33 (1.40)	3.03 (1.38)	2.65 (1.59)	2.54 (1.47)
EC2	3.22 (1.33)	2.99 (1.43)	2.65 (1.51)	2.48 (1.48)
Valence				
EC1	3.12 (1.28)	3.34 (1.17)	2.88 (1.24)	3.03 (1.35)
EC2	3.16 (1.24)	3.49 (1.33)	3.14 (1.54)	3.32 (1.44)
Calmness				
EC1	2.82 (1.33)	3.23 (1.33)	2.84 (1.47)	3.19 (1.49)
EC2	2.85 (1.32)	3.18 (1.24)	3.02 (1.32)	3.24 (1.25)
Perceived Strain				
EC1	2.07 (2.34)	2.30 (2.43)	1.93 (2.55)	1.93 (2.24)
EC2	1.80 (2.48)	1.70 (2.05)	1.51 (2.04)	1.30 (1.93)
**Exp. 1b**				
General rumination rating				
EC1	2.15 (2.56)	2.90 (3.19)	2.80 (3.10)	2.25 (2.53)
EC2	1.78 (1.85)	3.78 (2.45)	3.00 (2.69)	4.00 (2.55)
NCC	2.70 (3.46)	4.30 (4.05)	3.30 (3.92)	2.90 (3.28)
Energetic Arousal				
EC1	3.20 (1.47)	2.85 (1.40)	2.12 (1.29)	2.00 (1.23)
EC2	3.11 (1.08)	3.22 (0.87)	2.50 (1.03)	2.44 (1.16)
NCC	3.20 (1.73)	3.05 (1.72)	2.65 (1.49)	2.90 (1.98)
Valence				
EC1	3.22 (1.39)	2.67 (1.45)	2.42 (1.53)	2.52 (1.54)
EC2	2.89 (1.17)	3.05 (0.95)	3.05 (0.92)	3.44 (0.73)
NCC	3.45 (1.48)	2.90 (1.95)	2.60 (1.71)	3.00 (1.82)
Calmness				
EC1	2.55 (1.51)	2.77 (1.53)	2.57 (1.53)	3.02 (1.61)
EC2	3.00 (1.00)	2.83 (1.00)	2.67 (1.25)	3.39 (0.78)
NCC	2.85 (0.94)	2.75 (1.16)	2.55 (1.32)	3.10 (1.66)
Perceived Strain				
EC1	2.60 (2.72)	2.90 (3.19)	2.20 (2.67)	1.75 (2.40)
EC2	1.71 (2.22)	3.78 (2.44)	2.22 (1.99)	2.33 (2.00)
NCC	2.40 (3.17)	4.30 (4.05)	3.10 (3.66)	2.40 (3.24)

Table S3 continued

	SART measures			
	t_1	t_2	t_3	t_4
Exp. 1c				
Ruminative self-focus				
EC1	2.95 (1.88)	3.30 (1.71)	2.92 (1.92)	2.80 (1.67)
EC2	2.27(1.28)	2.52 (1.51)	2.37 (1.39)	2.25(1.61)
NCC	2.90 (1.39)	2.77 (1.54)	2.85 (1.40)	2.60 (1.20)
Energetic Arousal				
EC1	3.10 (1.48)	2.27 (1.66)	2.45 (1.71)	2.42 (1.79)
EC2	3.35 (1.48)	2.77 (1.53)	2.02 (1.47)	1.82 (1.32)
NCC	2.92 (1.41)	2.60 (1.38)	2.75 (1.48)	2.47 (1.37)
Valence				
EC1	2.90 (1.43)	2.82 (1.66)	2.42 (1.64)	2.65 (1.83)
EC2	3.15 (1.10)	2.72 (1.38)	2.45 (1.29)	2.37 (1.24)
NCC	3.40 (1.51)	3.32 (1.45)	3.20 (1.33)	3.27 (1.12)
Calmness				
EC1	3.02 (1.41)	3.10 (1.16)	2.95 (1.44)	3.02 (1.45)
EC2	2.57 (1.50)	2.55 (1.55)	2.37 (1.85)	2.67 (1.81)
NCC	3.32 (1.63)	3.50 (1.61)	3.42 (1.18)	3.37 (1.28)
Perceived Strain				
EC1	2.70 (1.84)	2.95 (1.70)	2.60 (1.90)	2.65 (1.87)
EC2	2.25 (1.71)	2.20 (1.64)	1.95 (1.43)	2.00 (1.41)
NCC	2.15 (1.27)	2.30 (1.62)	2.25 (1.45)	1.70 (0.80)

Note. NCC = neutral control condition 2, EC = experimental condition, t_1 = time point 1 (SARTbreak_1), t_2 = time point 2 (SARTblock_1), t_3 = time point 3 (SARTbreak_2), t_4 = time point 4 (SARTblock_2)
