# Supplementary material for: Experimental induction of state rumination: A study evaluating the efficacy of goal-cueing task in different experimental settings
Source: PLoS One. 2023 Nov 22;18(11):e0288450. doi: 10.1371/journal.pone.0288450 (PMC10664951; doi:10.1371/journal.pone.0288450)
Supplement: S4 Table — (PDF) [file pone.0288450.s004.pdf]

Table S4

Results of the multivariate analysis with group, gender and measure time as well as their respective interaction as factors and different rumination measures as dependent variables for Experiment 2.

	df	*F*-value	*p*-value	ηp²
**BSRI**				
group	3,195	2.49	.06	.04
gender	1,195	2.91	.09	.01
measure_time	1,195	10.77	.001	.05
group:gender	3,195	< 1	.70	< .01
group:measure_time	3,195	7.18	< .001	.10
gender:measure_time	1,195	< 1	.67	< .01
group:gender:measure_time	3,195	<1	.83	< .01
**MRNT**				
group	3,195	3.73	.01	.05
gender	1,195	6.61	.01	.03
measure_time	1,195	2.54	.11	.01
group:gender	3,195	< 1	.90	<.01
group:measure_time	3,195	9.71	< .001	.13
gender:measure_time	1,195	1.80	.18	< .01
group:gender:measure_time	3,195	< 1	.57	.01
**Ruminative self-focus**				
group	3,195	14.12	< .001	.18
gender	1,195	3.34	.07	.02
Measure_time	1,195	4.98	.03	.02
group: gender	3,195	< 1	.99	< .01
group:measure_time	3,195	26.85	< .001	.29
gender:measure_etime	1,195	< 1	.51	< .01
group:gender:measure_time	3,195	< 1	.63	<.01
**General rumination rating**				
group	3,195	7.02	< .001	.10
gender	1,195	3.01	.08	.01
Measure_time	1,195	< 1	.87	< .01
group: gender	3,195	< 1	.67	< .01
group:measure_time	3,195	12.21	< .001	.16
gender:measure_etime	1,195	< 1	.35	< .01
group:gender:measure_time	3,195	1.33	.26	.02

Note. We excluded two participants with divers gender for the analyses. BSRI = Brief State Rumination Inventory (Marchetti et al., 2018); MRNT = Momentary repetitive negative thinking (Rosenkranz et al., 2020); *df* = degrees of freedom, ηp² = partial eta squared (effect size)
