# Supplementary material for: Experimental induction of state rumination: A study evaluating the efficacy of goal-cueing task in different experimental settings
Source: PLoS One. 2023 Nov 22;18(11):e0288450. doi: 10.1371/journal.pone.0288450 (PMC10664951; doi:10.1371/journal.pone.0288450)
Supplement: S5 Table — (PDF) [file pone.0288450.s005.pdf]

Table S5

Test statistics for variables during the SART and corresponding effect sizes (ηp^2) of the respective mixed ANOVAs separated by effects for Experiment 2.

	**Condition effect**		**Time effect**		**Interaction effect**	
	Results *F*-statistic	ηp^2	Results *F*-statistic	ηp^2	Results *F*-statistic	ηp^2
State Rumination						
Ruminative self-focus	$F(3,201) < .01$	-	$F(6,201) < .01$	-	$F(3,201) < .01$	-
General rumination rating	$F(3,201) = 1.42$, $p = .24$	.02	$F(6,201) = 5.12$, p[GG] < .01	.03	$F(3,201) < .01$	-
Mood						
Energetic Arousal	$F(3,201) =1.74$, $p = .16$	.02	$F(6,201) = 47.91$, p[GG] < .01	.19	$F(3,201) =1.85$, p[GG] = .06	.03
Valence	$F(3,201) =1.59$, $p = .19$	.02	$F(6,201) = 12.89$, p[GG] < .001	.06	$F(3,201) =1.41$, p[GG] = .19	.02
Calmness	$F(3,201) =2.79$, $p = .04$	.04	$F(6,201) = 1.29$, p[GG] =.28	<.01	$F(3,201) < .01$	-
Perceived strain	$F(3,201) < .01$	-	$F(6,201) < .01$	-	$F(3,201) < .01$	-

Note. Exp. = experiments, *p[GG]* = *p*-value was corrected with Greenhouse-Geisser correction.
